# Nanosized Anatase TiO_2_ with Exposed (001) Facet for High-Capacity Mg^2+^ Ion Storage in Magnesium Ion Batteries

**DOI:** 10.1007/s40820-025-01861-7

**Published:** 2025-08-01

**Authors:** Rong Li, Liuyan Xia, Jili Yue, Junhan Wu, Xuxi Teng, Jun Chen, Guangsheng Huang, Jingfeng Wang, Fusheng Pan

**Affiliations:** 1 National Engineering Research Center for Magnesium Alloys, National Innovation Center for Industry-Education Integration of Energy Storage Technology, College of Materials Science and Engineering, Chongqing University, Chongqing, 400044 People’s Republic of China; 2 Chongqing Institute of New Energy Storage Materials and Equipment, Chongqing, 401135 People’s Republic of China; 3 Department of Materials, University of Oxford, Oxford, OX1 3PH UK

**Keywords:** Magnesium ion batteries, High capacity, Nanosized anatase TiO_2_, Crystal facet, Interfacial ion storage

## Abstract

Micro-sized anatase TiO_2_ displays inferior capacity as cathode material for magnesium ion batteries because of the higher diffusion energy barrier of Mg^2+^ in anatase TiO_2_ lattice. Herein, we report that nanosized anatase TiO_2_ exposed (001) facet doubles the capacity compared to the micro-sized sample ascribed to the interfacial Mg^2+^ ion storage. First-principles calculations reveal that the diffusion energy barrier of Mg^2+^ on the (001) facet is significantly lower than those in the bulk phase and on (100) facet, and the adsorption energy of Mg^2+^ on the (001) facet is also considerably lower than that on (100) facet, which guarantees superior interfacial Mg^2+^ storage of (001) facet. Moreover, anatase TiO_2_ exposed (001) facet displays a significantly higher capacity of 312.9 mAh g^−1^ in Mg–Li dual-salt electrolyte compared to 234.3 mAh g^−1^ in Li salt electrolyte. The adsorption energies of Mg^2+^ on (001) facet are much lower than the adsorption energies of Li^+^ on (001) facet, implying that the Mg^2+^ ion interfacial storage is more favorable. These results highlight that controlling the crystal facet of the nanocrystals effectively enhances the interfacial storage of multivalent ions. This work offers valuable guidance for the rational design of high-capacity storage systems.

**Supplementary Information:**

The online version contains supplementary material available at 10.1007/s40820-025-01861-7.

## Highlights

- Nanosized anatase TiO_2_ exposed (001) facet doubles the capacity compared to the micro-sized sample ascribed to the interfacial Mg^2+^ ion storage.
- Anatase TiO_2_ exposed (001) facet displays a significantly higher capacity of 312.9 mAh g^−1^ in Mg–Li dual-salt electrolyte.
- The adsorption energies of Mg^2+^ on (001) facet are much lower than the adsorption energies of Li^+^ on (001) facet, implying that the Mg^2+^ ion interfacial storage is more favorable.

**Supplementary Information:**

The online version contains supplementary material available at 10.1007/s40820-025-01861-7.

## Graphical abstract

**Figure:**
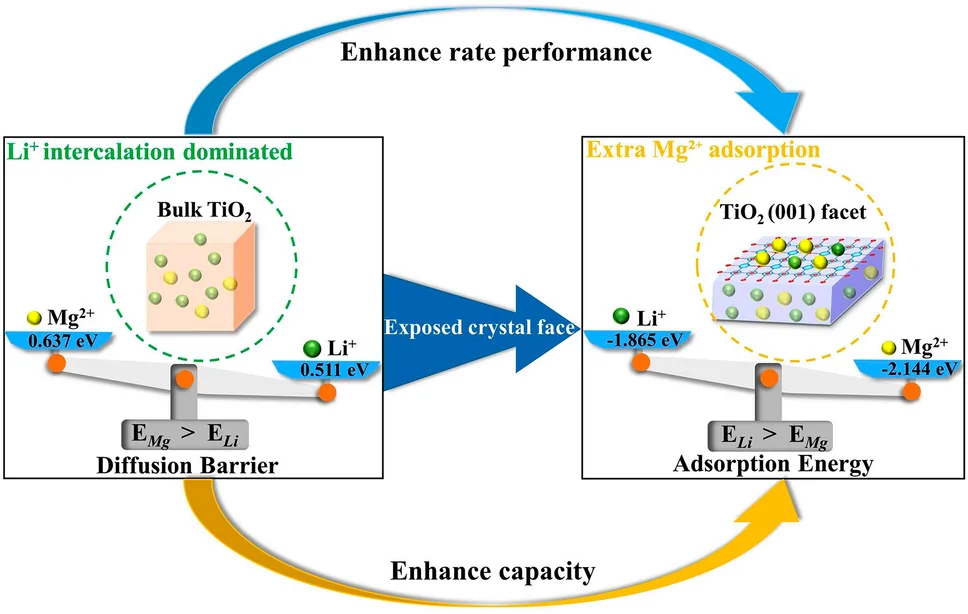

## Introduction

Nanostructure engineering becomes a revolutionary strategy to overcome the inherent limitations of bulk materials by precisely manipulating size effects and atomic arrangements on surfaces [1–4]. In the field of secondary batteries, the size and morphology of electrode materials are critical determinants of electrochemical performance [3, 5, 6]. Nanostructured materials reduce particle size, shortening the ion diffusion path, enhancing specific capacity and rate performance. Meanwhile, controlling the exposure of specific crystal facets in materials can provide more ion diffusion channels, significantly improving ion diffusion ability and thereby enhancing the ion storage capacity of electrode materials [2, 7–12]. Therefore, the combination of nanostructuring and controlled crystal facet exposure is a crucial strategy for enhancing the performance of electrode materials. Nanosized Li_4_Ti_5_O_12_ effectively shortens Li^+^ diffusion pathways, displayed a high specific capacity of ~ 175 mAh g^−1^ at ranges of 1–50 C (0.17–8.7 A g^−1^) [13]. Nanostructured T-Nb_2_O_5_ with (001) plane provides low energy barriers, facilitating rapid Li^+^ transport (60 meV) [14]. Furthermore, controlling the growth of the (010) plane during the synthesis of LiFePO_4_ can effectively enhance the Li^+^ diffusion [8, 15]. These examples highlight that nanostructuring and controlled exposure of specific crystal planes in electrode materials markedly improve ionic transport kinetics and rate capabilities [16–18].

The researches on nanostructuring and crystal facets control are currently primarily focused on monovalent Li^+^ systems. Compared to monovalent Li^+^, Mg^2+^ exhibits sluggish diffusion kinetics in electrode materials due to their higher charge density (120 vs. 52 C mm^−3^) [19–21]. This results in low reversible intercalation efficiency and poor energy density, significantly hindering the development of magnesium ion batteries (MIBs) [22–25]. To improve the diffusion kinetics of Mg^2+^, researchers have recently discovered that introducing Li^+^ into MIBs system and constructing a Mg–Li dual-salt ion batteries (MLIBs) can be effective [16, 26, 27]. The “charge shielding effect” induced by Li^+^ effectively mitigates the strong polarization effect of Mg^2+^, improving Mg^2+^ diffusion kinetics and to enhance the material energy density. This dual-salt system has become a widely adopted strategy for improving the performance of MIBs [28]. Compared to conventional ion system optimizations, nanostructured materials can fundamentally improve material properties, and their advantages are becoming increasingly evident [29, 30]. Nanostructured materials possessing a large specific surface area can effectively shorten the diffusion pathways for ions, providing fast kinetic performance for the insertion and extraction of Mg^2+^, thereby improving electrochemical performance. For example, Jiang et al*.* demonstrated that VO_x_ nanotubes achieved a capacity of 75 mAh g^−1^ for Mg^2+^ insertion in non-aqueous electrolytes [29]. Additionally, a combination of vanadium pentoxide (V_2_O_5_) and graphene nanoplatelets (GNPs) achieved a specific capacity of 90 mAh g^−1^ in MIBs [29]. Although these examples suggest that nanostructuring has some effect in improving the performance of MIBs, the enhancement in performance is not substantial. Moreover, these studies have not extensively explored the specific changes in Mg^2+^ diffusion kinetics following nanostructuring. Additionally, research on the influence of controlling specific crystal facets on Mg^2+^ diffusion and storage behavior is relatively limited, and this gap is particularly evident in MIBs. Therefore, we propose that nanostructuring with controlled exposure of specific crystal facets can enhance interfacial Mg^2+^ storage, thereby improving the capacity.

Herein, we find that by nanostructurally tuning the crystal facet exposure of anatase TiO_2_, the interfacial Mg^2+^ storage capability can be significantly enhanced. The (100) and (001) facets exhibit higher reactivity compared to the conventional (101) facet, providing better ion diffusion pathways [31]. Experimental results show that nanostructured TiO_2_ materials, especially those dominated by (001) crystal faces, significantly enhance Mg^2+^ storage and transport performance. Theoretical calculations reveal that the diffusion energy barrier for Mg^2+^ on the (001) and (100) facets is much lower than in the bulk phase. Additionally, adsorption energy calculations show that Mg^2+^ with the lowest adsorption energy on the (001) facet, which is also significantly lower than that for Li^+^ on the (001) facet. This indicates that the (001) facet significantly promotes Mg^2+^ storage, increasing the system capacity. Electrochemical test results show that the anatase TiO_2_ with the (001) crystal facet exposed exhibits the best electrochemical performance, followed by the (100) crystal facet, while the electrochemical performance of the bulk TiO_2_ is the lowest. In pure Li salt electrolyte, the capacity increase for the (001) facet exposure (70.77%) is significantly lower than that in Mg salt electrolytes (90.24%), indicating a limited interfacial Li^+^ storage effect. The enhanced capacity in the dual-salt electrolyte system is primarily attributed to the interfacial storage of Mg^2+^. These results confirm that controlling the nanocrystal facet exposure can effectively enhance the interfacial storage of multivalent ions. Therefore, nanostructural tuning of facet exposure is an effective strategy to enhance battery performance, particularly in improving the capacity and rate performance of multivalent ion batteries. This study provides a new horizon for the nanoscale effects of electrode materials and offers valuable guidance for the rational design of high-capacity and high-rate energy storage systems.

## Experimental Section

### Materials Preparation

#### Synthesis of Anatase (001) Nanosheets

In a 50 mL dry PTFE (polytetrafluoroethylene) high-pressure reaction vessel, 5 mL of titanium tetrabutoxide (Ti(OBu)_4_) was thoroughly mixed with 0.8 mL of hydrofluoric acid (HF) solution. The reaction vessel was maintained at 200 °C for 24 h. After the reaction, the vessel was allowed to cool to room temperature, and the powder was separated by high-speed centrifugation. The product was then washed 4–5 times with ethanol and distilled water. Finally, the resultant product was dried in a vacuum oven at 60 °C for 24 h, yielding anatase TiO_2_ nanosheets with exposed (001) crystal facets.

#### Synthesis of Anatase (100) Involves Two Steps

Step 1: 2 g of P25 was added to 80 mL of 10 M sodium hydroxide (NaOH) aqueous solution and thoroughly stirred before transferring the mixture to a 100 mL PTFE-lined stainless-steel high-pressure reaction vessel. The reaction was conducted at 120 °C for 24 h, after which the vessel was cooled to room temperature. The white sodium titanate (Na_2_TiO_3_) precipitate was separated by high-speed centrifugation and washed repeatedly with deionized water until the pH of the solution stabilized at approximately 10.5. Step 2: 1 g of the sodium titanate precipitate obtained from Step 1 (without drying) was added to 40 mL of deionized water. The mixture was then transferred to a 50 mL PTFE-lined stainless-steel high-pressure reaction vessel and heated at 200 °C for 24 h. After the reaction, the white precipitate was separated by high-speed centrifugation and washed 4–5 times with deionized water. Finally, the product was dried in a vacuum oven at 60 °C for 24 h, yielding anatase TiO_2_ nanorods with exposed (100) crystal facets.

#### Commercial Anatase TiO_2_

The commercially available TiO_2_ (Sigma-Aldrich, particle size < 20 nm) was used as the starting material in this work.

All reagents used in this study were employed directly after purchase without any additional treatment.

### Characterization

The crystal structure of the samples was determined by X-ray diffraction (XRD, Rigaku Ultima IV). The microstructure and morphology of the samples were characterized using scanning electron microscopy (SEM, JEOL JSM-7800F) and transmission electron microscopy (TEM, JEOL JEM-F200). High-resolution transmission electron microscopy (HRTEM) and energy dispersive spectroscopy (EDS-mapping) were used to analyze the phase structure and chemical element distribution of the materials. X-ray photoelectron spectroscopy (XPS, ESCALAB 250Xi) was performed using a Thermo Scientific K-Alpha instrument. The specific surface area and porosity characteristics of the materials were measured using the Brunauer–Emmett–Teller (BET) method with a Quantachrome Instruments surface area analyzer.

### Electrochemical Measurement

The preparation of the APC electrolyte is referenced from previous reports. Typically, 1.0067 g of AlCl_3_ is slowly added to 12 mL of tetrahydrofuran (THF) and stirred for 12 h. Then, 12 mL of THF is mixed with 8 mL of PhMgCl, and the two solutions are stirred for 12 h to obtain a 0.4 M APC electrolyte. A 0.4 M LiCl solution is then added to the 0.4 M APC electrolyte and stirred overnight to prepare a magnesium–lithium mixed electrolyte. All preparation steps are carried out in an argon-filled glove box. A mixture of 70 wt% sample material, 20 wt% Super P, and 10 wt% polyvinylidene fluoride (PVDF) is thoroughly ground and then stirred with* N*-methyl-2-pyrrolidone (NMP) at room temperature for 8 h. The resulting slurry is coated onto carbon paper, blow-dried, and then vacuum-dried at 80 °C before being punched into 12 mm diameter disks for use as the cathode. Electrochemical performance is tested using a 2032 coin-type cell with freshly polished Mg as the anode. The electrochemical tests are performed in the voltage range of 0.1–2.0 V versus Mg^2+^/Mg. Full charge–discharge tests are conducted using a Neware battery testing system, with the cells being rested for 10 h before testing. Cyclic voltammetry measurements are carried out using a CHI electrochemical workstation. All electrochemical measurements are performed at room temperature.

### Computational Methods

The calculations were conducted using the Vienna Ab initio Simulation Package (VASP), employing density functional theory (DFT) for all simulations [32]. The Perdew–Burke–Ernzerhof (PBE) generalized gradient approximation (GGA) was used for the exchange–correlation functional [33, 34]. The plane-wave cutoff energy was set to 520 eV, ensuring accurate representation of the wave functions. Energy convergence was set at 10^–6^ eV, while geometry optimizations were carried out until the forces on each atom were less than 0.02 eV Å^−1^. Two surface models, anatase TiO_2_ with exposed (001) and (100) crystal facets, were constructed using a 2 × 2 supercell configuration, each containing 16 Ti atoms and 32 O atoms, maintaining a Ti:O atom ratio of 1:2 consistent with the bulk structure. A vacuum layer of 15 Å was introduced to prevent interactions between periodic images. The k-point grid for sampling was set to 2 × 3 × 1 for the (001) facets model and 3 × 2 × 1 for the (100) facets model, using the Monkhorst–Pack scheme [35]. For diffusion energy barriers calculations, the climbing image nudged elastic band (CI-NEB) method was employed [36]. Additionally, structural optimizations incorporated Grimme’s third-generation (D3) van der Waals (vdW) corrections to account for dispersion forces and improve the accuracy of the simulation [37, 38]. This comprehensive approach ensures the reliability of the calculated energy barriers and structural characteristics.

## Results and Discussion

### Materials Characterization

**Fig. 1:**
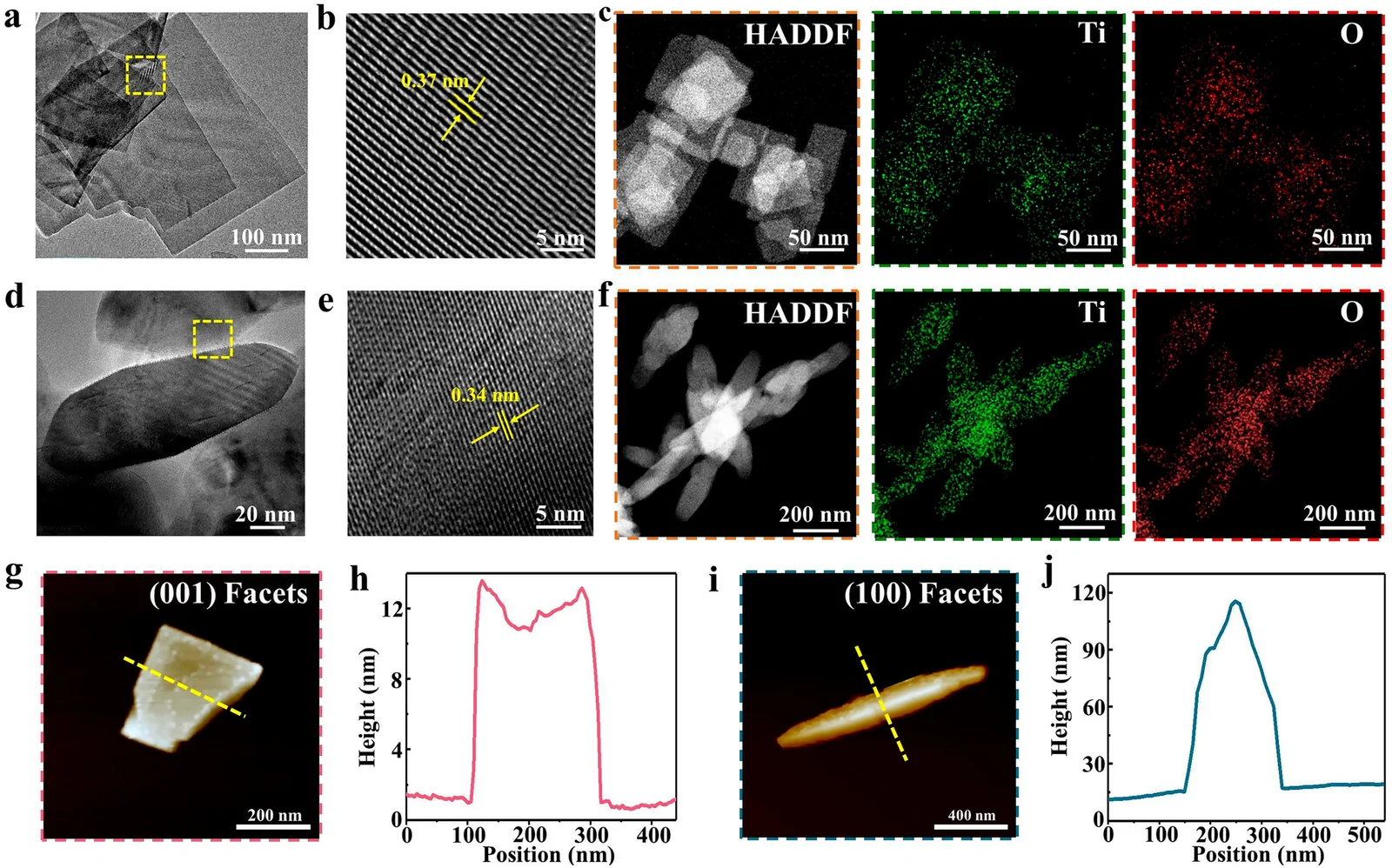
**a**, **b** TEM and HRTEM images of TiO_2_ (001) facet. **c** HADDF image and elemental mappings of TiO_2_ (001) facet. **d**–**e** TEM and HRTEM images of TiO_2_ (100) facet. **f** The HADDF image and elemental mappings of TiO_2_ (100) facet. **g**, **h** AFM image and height profiles of TiO_2_ (001) facet and **i**, **j** TiO_2_ (100) facet along the white dashed lines

The TiO_2_ nanosheets with exposed (001) facet and nanorods with exposed (100) facet were synthesized via a simple hydrothermal method, and the synthesis process is shown in Fig. S1. XRD patterns of the synthesized TiO_2_ samples, shown in Fig. S2, reveal diffraction peaks that can be indexed to the anatase phase TiO_2_ (JCPDS No. 21-1272), confirming the high phase purity of the obtained materials. SEM images (Fig. S3) provide a direct visualization of the morphological characteristics of the two materials. The TiO_2_ with exposed (001) facet exhibits a typical nanosheet structure. TEM images in Fig. 1a further confirm that the (001)-facet-exposed TiO_2_ consists of rectangular nanosheets with an average edge length of approximately 40 nm. High-resolution TEM (HRTEM) imaging (Fig. 1b) reveals well-defined lattice fringes with an interplanar spacing of 0.372 nm, corresponding to the (001) plane of anatase TiO_2_, further verifying the facet-oriented nature of the material. As shown in Fig. S3e, f, the morphological features of the (100)-facet-exposed nanorods are displayed in Fig. S2e, f, showing uniform and well-aligned tetragonal nanorods. Their lateral surfaces are primarily composed of four highly active (100) facet with sharp edges and uniform widths. Different from the regular (001) and (100) facets, the original anatase TiO_2_ exhibits a rough and irregular particle aggregation morphology (Fig. S3i, j), lacking ordered crystal facet exposure. Particle aggregation usually leads to a reduction in the effective surface area available for electrochemical reactions in ion battery, which adversely affects the battery’s capacity and charge–discharge performance. Additionally, particle aggregation also leads to decrease in ion transport rate. Thus, optimizing these morphological features, reducing particle aggregation, and improving particle uniformity are crucial for enhancing the overall performance of lithium–ion batteries. HRTEM images (Fig. 1d, e) reveal a crystal face spacing of 0.338 nm in the transverse direction. The brightness profiles extracted from the HRTEM images of both materials are provided in Fig. S4. Furthermore, elemental mapping images demonstrate that Ti and O elements are homogeneously distributed within the synthesized TiO_2_ samples with exposed (001) and (100) facet, confirming their uniform chemical composition and phase purity (Fig. 1c, f). Additionally, atomic force microscopy (AFM) measurements determined that the thickness of the (001)-facet-exposed nanosheets is approximately 12 nm (Fig. 1g, h), while the (100)-facet-exposed nanorods are significantly thicker, measuring around 120 nm (Fig. 1i, j). To precisely assess the differences in specific surface area and porosity between the (001)-and (100)-facet-exposed materials, Brunauer–Emmett–Teller (BET) measurements were performed (Fig. S5). The results indicate that the (001)-facet-exposed TiO_2_ exhibits a high specific surface area of 126 m^2^ g^−1^, a pore size of 20.24 nm, and a pore volume of 0.62 cm^3^ g^−1^. This mesoporous structure facilitates abundant diffusion pathways for Mg^2+^ transport, thereby promoting rapid Mg^2+^ diffusion and enhancing cycling stability. In comparison, the (100)-facet-exposed TiO_2_ nanorods also exhibit a mesoporous nature but with a slightly lower specific surface area of 67 m^2^ g^−1^, a pore size of 14.98 nm, and a pore volume of 0.21 cm^3^ g^−1^. The structural characteristics resulting from facet exposure play a crucial role in optimizing Mg^2+^ storage and transport properties.

### Theoretical Calculation-Ion Adsorption/Diffusion

**Fig. 2:**
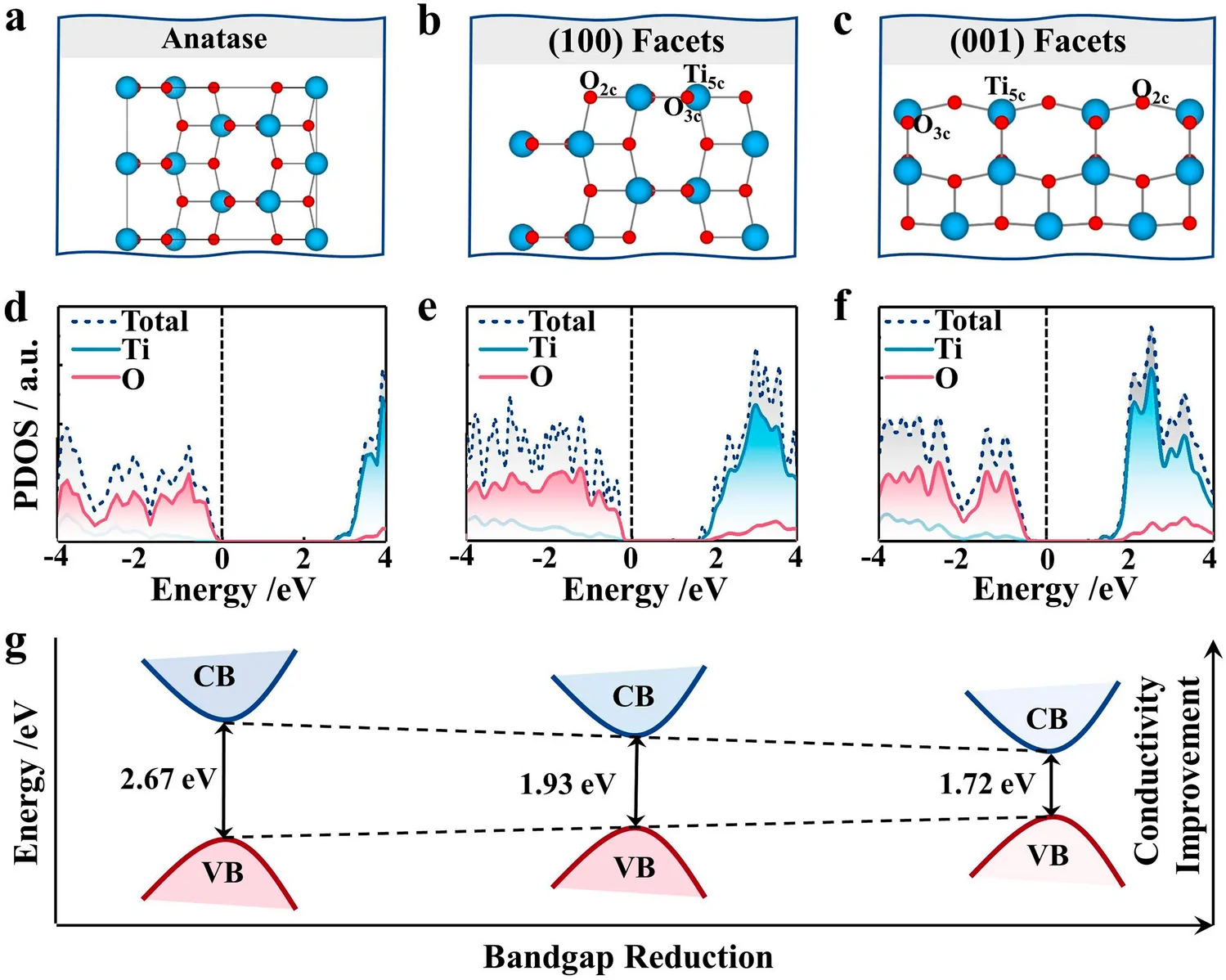
**a**–**c** Structures of anatase TiO_2_, TiO_2_ (001) facet and (100) facet. **d**–**f** corresponding DOS of anatase TiO_2_, TiO_2_ (001) facet and (100) facet. **g** Change of band gap of three kinds of materials

To systematically elucidate the impact of different exposed facets on the electrochemical performance of anatase TiO_2_ in both pure Mg and Mg–Li dual ion systems, we conducted a series of DFT calculations and experimental studies. These investigations aimed to explore the mechanistic influence of facet effects on the electronic structure and Mg storage behavior of the material. As previously discussed, exposing specific crystal facets can significantly enhance electrochemical performance by increasing the specific surface area, which not only facilitates ion diffusion but also improves electronic properties, increases ion storage sites, and promotes subsequent ion intercalation and de-intercalation. To gain deeper insights into this phenomenon, we constructed slab models of (001) and (100)-facet-exposed TiO_2_ (Fig. 2a–c) and performed DFT calculations to evaluate ions adsorption. First, the density of states (DOS) was calculated for three structures, bulk TiO_2_, (001)-facet-exposed TiO_2_, and (100)-facet-exposed TiO_2_. Analysis based on the Perdew–Burke–Ernzerhof (PBE) functional indicates that compared to bulk TiO_2_ (bandgap: 2.15 eV), the bandgaps of the (001) and (100)-facet-exposed TiO_2_ decrease to 1.78 and 1.92 eV, respectively (Fig. 2d–f), demonstrating that facet exposure effectively modulates the electronic structure. To experimentally validate these computational results, ultraviolet–visible diffuse reflectance spectroscopy (UV–Vis DRS) combined with the Tauc extrapolation method was employed (Fig. S6). The measured bandgap of pristine anatase TiO_2_ was found to be 3.24 eV, which decreased to 2.83 and 3.05 eV upon exposure of the (001) and (100) facets, respectively. The observed experimental trends are in excellent agreement with the relative changes predicted by DFT (Fig. 2g), despite the well-known tendency of the PBE functional to systematically underestimate absolute bandgap values due to its neglect of exact exchange interactions. It is important to emphasize that the primary objective of using the PBE functional in this study was to efficiently screen trends in electronic property variations among different exposed facets. The strong correlation between computational and experimental results confirms the validity of this approach. This modulation of electronic structure also manifests in distinct macroscopic optical properties. As shown in Fig. S7, the (001)-facet-exposed sample exhibits a blue appearance, whereas the (100)-facet-exposed sample appears nearly white, like pristine TiO_2_, which remains characteristically white. This observation further supports the role of facet engineering in tuning the electronic structure and electrical conductivity. A narrower bandgap enhances electronic conductivity, improving charge carrier mobility and accelerating charge transfer at the electrode/electrolyte interface. This, in turn, facilitates ion intercalation and de-intercalation kinetics, thereby enhancing the reversible storage capability of Mg^2+^.

**Fig. 3:**
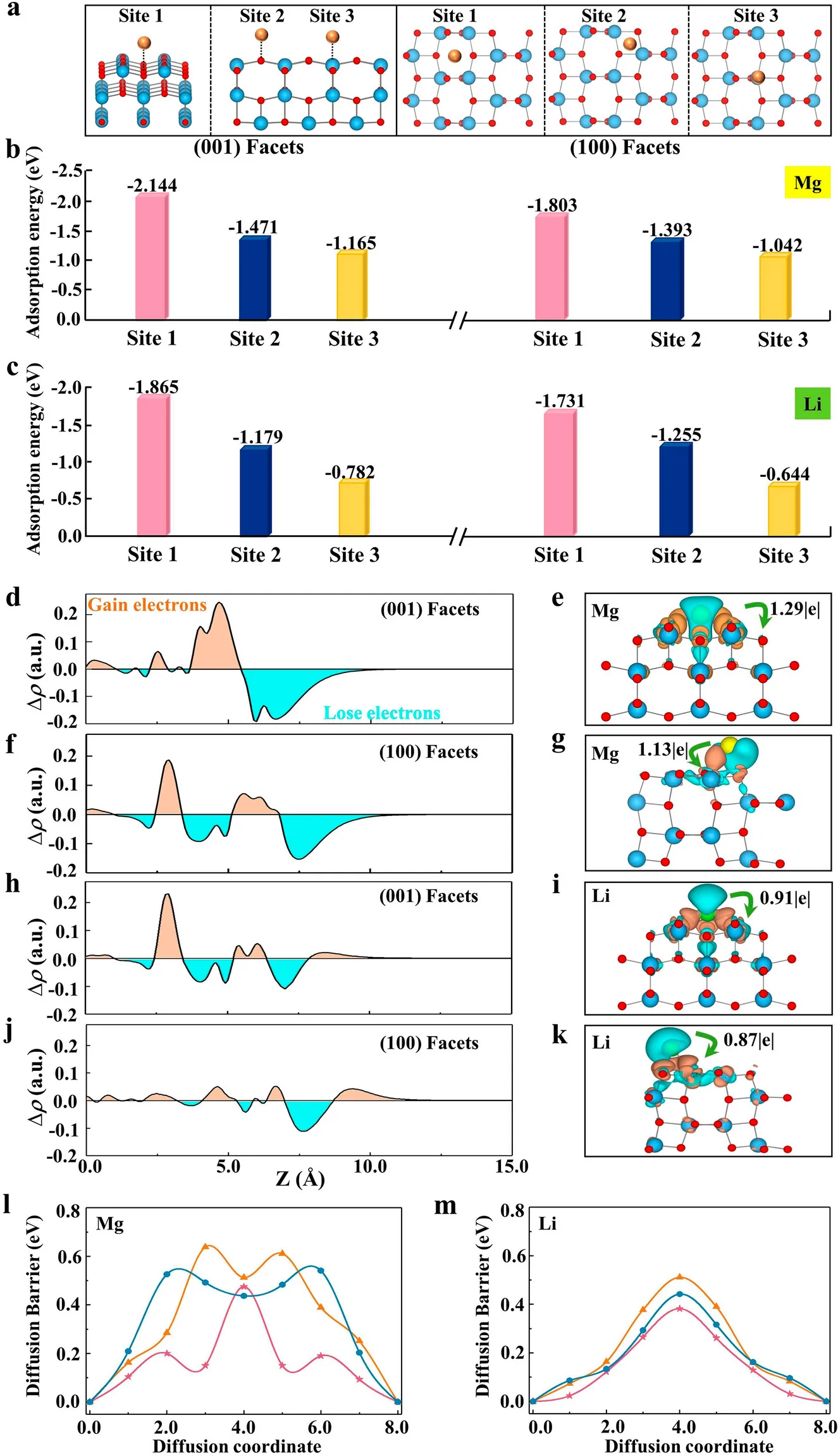
**a** Schematic illustration of Mg^2+^ and Li^+^ adsorption sites on the TiO_2_ (001) facet and (100) facets, with different adsorption sites labeled as Site 1, Site 2, and Site 3. **b** Adsorption energies of Mg^2+^ at different adsorption sites on the TiO_2_ (001) and (100) facets. **c** Adsorption energies of Li⁺ at different adsorption sites on the TiO_2_ (001) facet and (100) facets. **d**, **e** The planar average charge density difference Δρ(z) of Mg^2+^ adsorption on the TiO_2_ (001) facet and the three-dimensional charge density difference graph. The planar average charge density difference Δρ(z) and three-dimensional charge density difference graph of Mg^2+^ adsorption on the **f, g** TiO_2_ (100) facet. The planar average charge density difference Δρ(z) and three-dimensional charge density difference graph of Li^+^ adsorption on the **h, i** TiO_2_ (001) facet. The planar average charge density difference Δρ(z) and three-dimensional charge density difference graph of Li⁺ adsorption on the **j, k** TiO_2_ (100) facet. **l** Diffusion energy barrier of the Mg^2+^ and **m** Li⁺ on anatase TiO_2_ and TiO_2_ (001) facet and (100) facet. Triangle: anatase, circle: (100) facet, pentagram: (001) facet

To further investigate the impact of different exposed facets on ions storage behavior, we conducted systematic DFT calculations to evaluate the Mg^2+^ and Li^+^ adsorption affinity on various crystal facets. Figure 3a illustrates the possible adsorption sites on the (001) and (100) facets. The adsorption energies of Mg^2+^ (Fig. 3b) and Li⁺ (Fig. 3c) at different adsorption sites on the (001) and (100) surfaces were calculated accordingly. The results indicate that the (001) facet exhibits the lowest adsorption energy for both Mg^2+^ and Li⁺ (− 2.144 and − 1.865 eV), with Mg^2^⁺ exhibiting a significantly lower adsorption energy than Li⁺. In addition, considering that the most common exposed facet of pristine anatase TiO_2_ is the (101) crystal facet, we also constructed a slab model of the exposed (101) crystal facet and conducted surface ion adsorption energy calculations, as shown in Fig. S8. The calculated results show that the adsorption energies of Mg^2+^ and Li^+^ ions on the (101) surface are significantly higher than those on the (001) and (100) surfaces. This suggests that the exposed crystal facet strongly favors the adsorption of both ions, with the (001) facet displaying a notably higher affinity for Mg^2+^. Additionally, charge density difference (CDD) analysis and Bader charge analysis were performed to elucidate the role of facet exposure in ion adsorption and charge transfer mechanisms in Fig. 3b, c. Figure 3d, f presents the plane-averaged charge density difference Δρ(z) along the vertical (z) axis, computed as the CDD between the substrate and the adsorbed Mg atom. A positive Δρ(z) indicates charge accumulation, whereas a negative value represents charge depletion. The results reveal that Mg loses electrons on both facets, accompanied by significant electron transfer to the substrate (cyan regions), while the substrate surface gains electrons (orange regions). This phenomenon is further illustrated in the three-dimensional charge density difference plots, which clearly depict charge gain and loss upon Mg adsorption (Fig. 3e, g). To quantify the degree of charge transfer on different facets, Bader charge analysis was performed. The results show that the charge transfer amount on the (001) facet is larger (1.29|e| vs. 1.13|e| for the (100) facet), confirming the stronger electronic attraction of the (001) facet toward Mg ions. Additionally, similar charge density difference and Bader charge calculations were conducted for Li ion adsorption on both surfaces as shown in Fig. 3h–k. The computed charge transfer values for Li adsorption are 0.91|e| for the (001) facet and 0.87|e| for the (100) facet. These findings indicate that the (001) facet exhibits a higher affinity for both Mg^2^⁺ and Li⁺, potentially offering greater stability and stronger bonding interactions, thereby enhancing the reversible storage performance of Mg^2+^. Furthermore, the larger specific surface area introduced by facet exposure provides additional high-activity adsorption sites, directly contributing to the overall enhancement of ion storage capacity.

Ion diffusion kinetics play a decisive role in the energy storage performance of electrode materials, and the crystal facet structure directly influences ion migration behavior at both the interface and within the bulk. To systematically investigate the effect of specific facet exposure on the diffusion kinetics of Mg^2+^ and Li^+^, we performed climbing image nudged elastic band (CI-NEB) calculations to analyze the diffusion energy barriers of Mg^2+^ and Li^+^ on different facets and as well as within the pristine TiO_2_ lattice and elucidate the critical role of facet engineering in optimizing ion transport. The DFT-calculated diffusion activation barriers along migration pathways for Mg^2+^ and Li^+^ on different facets and in anatase TiO_2_ are presented in Fig. 3l, m. We considered two distinct diffusion pathways for Mg^2+^ and Li^+^ on the (001) and (100) facets to determine the most favorable ion migration pathways labeled path1 and path2. Here, we discuss the diffusion behavior of both ions along the optimal diffusion pathways path 1on the (001) and (100) facets as well as in anatase TiO_2_. The detailed ion diffusion paths and the corresponding diffusion energy barriers along the path 2 are presented in the Supplementary Information (Figs. S9 and S10). On the (001) facet, the diffusion barriers for Mg^2+^ along different paths are 0.475 eV ((001) facets), 0.542 eV ((100) facet), and 0.637 eV (anatase TiO_2_). The diffusion barriers for Li^+^ are 0.381 eV ((001) facet), 0.442 eV ((100) facet), and 0.511 eV (anatase TiO_2_). For both ions, these diffusion barriers are significantly lower than those in the bulk material. Mainly because the highly symmetrical and dense crystal structure in the bulk material limits the ion migration path, especially for Mg^2+^ with a higher charge density, its strong coulomb interaction with the surrounding oxygen atoms further hinders diffusion. Diffusion path diagram of Mg^2+^ and Li^+^ ions in the original anatase TiO_2_ is shown in Fig. S11. After exposing the crystal facets, shortens the ion diffusion channels, is more conducive to ion migration, and significantly reduces the diffusion energy barrier. These results clearly demonstrate that facet engineering effectively reduces surface diffusion energy barriers, with the (001) facet providing a more favorable kinetic environment for the diffusion of both Mg^2+^ and Li^+^. This finding confirms that exposing specific facets enhances ion migration rates at the material surface and facilitates ion entry into the bulk at the interface, thereby improving overall ion transport efficiency. Furthermore, the (001) facet, which exhibits a larger specific surface area, further enhances interfacial ion diffusion capability.

### Electrochemical Performance

**Fig. 4:**
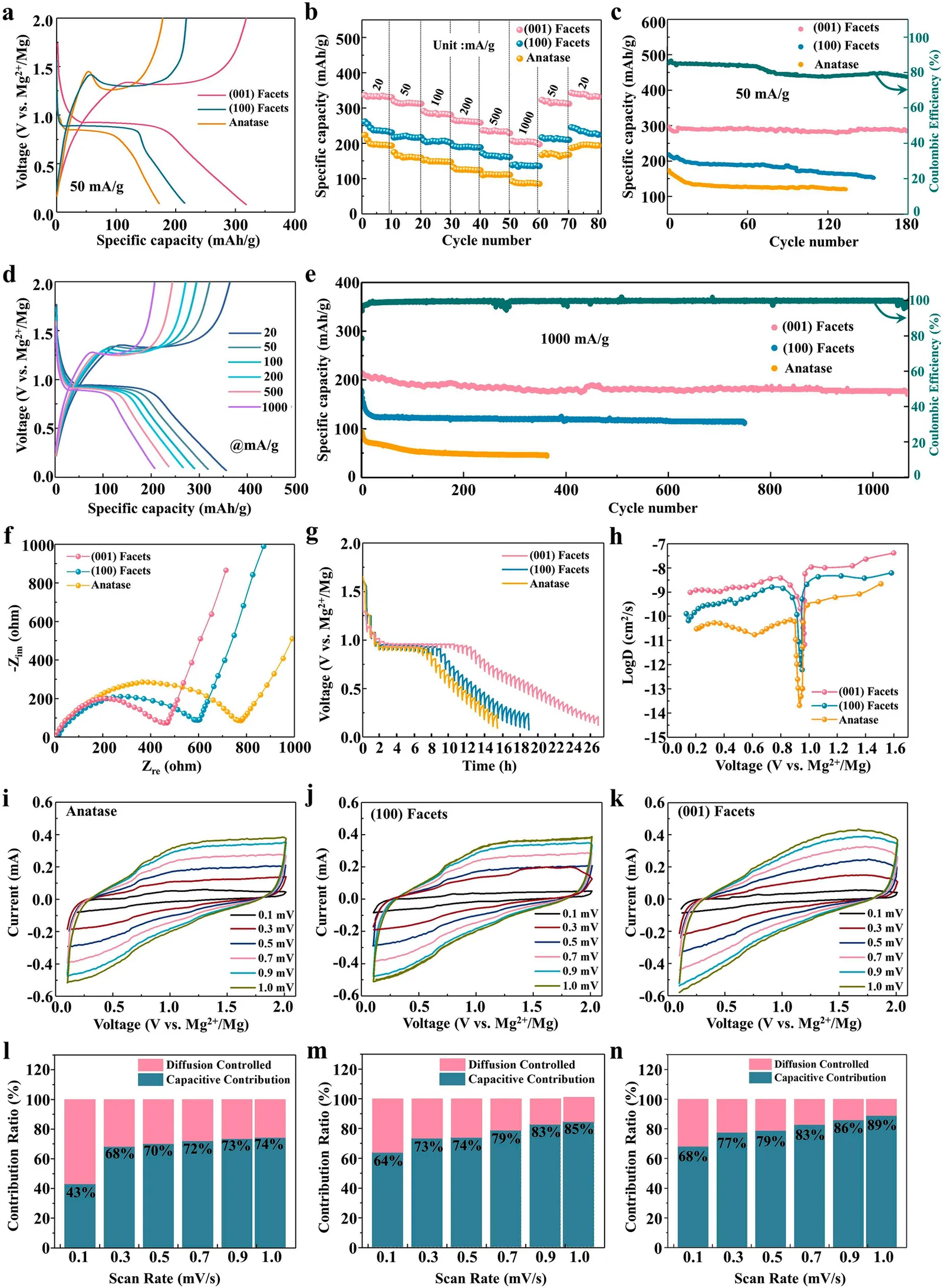
**a** Charge–discharge curves of anatase TiO_2_, TiO_2_ (001) facet, and TiO_2_ (100) facet at 50 mA g^−1^ in MLIBs.** b** Rate performance of anatase TiO_2_, TiO_2_ (001) facet and (100) facet in MLIBs. **c** Cycling capabilities of the TiO_2_ (001) facet at 50 mA g^−1^ in MLIBs. **d** Charge–discharge curves of the TiO_2_ (001) facet at current densities ranging from 20 mA g^−1^ to 1000 mA g^−1^ in MLIBs. **e** Cycling performances of the TiO_2_ (001) facet at 1000 mA g^−1^ in MLIBs. **f** Nyquist plots of anatase TiO_2_, TiO_2_ (001) facet and (100) facet in MLIBs. **g** GITT profiles for anatase TiO_2_, TiO_2_ (001) facet and (100) facet in MLIBs.** h** Calculated ions diffusion coefficients in MLIBs. **i**–**k** CV curves of anatase TiO_2_, TiO_2_ (001) facet and (100) facet at different scan rates in MIBs. **l**–**n** Capacitive contributions of anatase TiO_2_, TiO_2_ (001) facet and (100) facet at different scan rates in MIBs

Rate capability and cycling stability are critical indicators for evaluating the practical application potential of electrode materials. The electrochemical performance of pristine TiO_2_, as well as TiO_2_ with exposed (001) and (100) facets, was systematically investigated as cathode materials for Mg and Mg–Li hybrid batteries. Full-cell performance evaluations were conducted for assembled Mg and Mg–Li dual-salt batteries. Figure 4a presents the charge–discharge curves of pristine TiO_2_ and exposed (001) and (100) facets in Mg–Li dual-salt electrolyte at current density of 50 mA g^−1^. The results indicate that the (001) facet exhibits the highest specific capacity, reaching approximately 312.9 mAh g^−1^, which is significantly higher than that of the (100) facet material (215.8 mAh g^−1^) and pristine anatase (172.23 mAh g^−1^), demonstrating the significant role of the (001) facets in enhancing ion storage behavior. In addition, the charge–discharge curves presented in Fig. 4a clearly demonstrate significant differences in the voltage profiles of the three materials in the Mg–Li dual-salt electrolyte. Specifically, the (001) facet exhibits a relatively flat and stable voltage profile, indicating more efficient ion migration during the charge–discharge process. The (100) facet followed. In contrast, the pristine anatase material displays a steeper voltage change, particularly during the charging process, where a distinct voltage plateau peak is observed around 1.5 V. This phenomenon is likely related to the surface structure of the electrode material and its ion intercalation/de-intercalation behavior during charging. The appearance of this peak suggests that the pristine material suffers from poor ion transport and voltage regulation capabilities in the initial stages of charging, due to structural limitations that hinder reversible ion de-intercalation, leading to voltage fluctuations in the early charge–discharge cycles. In contrast, the (001) facet, owing to its unique exposed surface structure, can more effectively accommodate and release ions, resulting in higher specific capacity and a more stable voltage response.

To further elucidate the contribution of ionic species to the enhanced capacity, comparative electrochemical measurements were conducted in pure Li electrolyte. The results reveal that the (001) facet still delivers the highest capacity. Although the (001) facet exhibits improved capacity relative to pristine anatase TiO_2_ in the pure Li electrolyte (97.05 mAh g^−1^) as shown in Fig. S12, the increment is substantially lower than that observed in the dual-salt electrolyte (140.67 mAh g^−1^). Notably, both (001) and (100) facets demonstrate greater capacity enhancement across different electrolyte systems, indicating that the exposed facet enhances surface adsorption of metal ions. While both Li^+^ and Mg^2+^ can be adsorbed on the surface, the combined comparison of capacity increments and the calculated adsorption energies on exposed facet clearly indicates that Mg^2+^ exhibits a more favorable (more negative) adsorption energy. These results collectively suggest that the significant capacity increase in the dual-salt system primarily stems from the effective adsorption and utilization of Mg^2+^. This finding highlights the critical role of facet engineering in promoting Mg^2+^ storage and enhancing overall electrochemical performance. Moreover, the (001) facet material displays a relatively stable voltage plateau during charge–discharge cycles, with minimal polarization and reduced voltage hysteresis during discharge in MLIBs, indicating superior reaction kinetics and lower diffusion resistance. In contrast, the charge–discharge curve of the (100) facet material exhibits noticeable fluctuations with more pronounced voltage hysteresis. The pristine anatase TiO_2_ suffers from severe polarization effects, exhibiting significant voltage lag during charge–discharge processes. This suggests that its charge transport capability is insufficient, hindering the intercalation and de-intercalation of ions. Thus, the exposure of the (001) facet significantly enhances the ion storage capability and electrochemical reaction kinetics, leading to the best energy storage performance in MLIBs. Furthermore, in the Mg–Li dual-salt electrolyte, pristine anatase TiO_2_ exhibits the poorest rate capability among the three materials as shown in Fig. 4b. The material with exposed (001) facet demonstrates significantly superior rate performance and cycling stability. It maintains the highest specific capacity across all tested current densities (20–1000 mA g^−1^), with discharge capacities of 333.2, 312.9, 281.1, 262.3, 233.4, and 201.6 mAh g^−1^ at 20, 50, 100, 200, 500, and 1000 mA g^−1^, respectively. Additionally, although the discharge capacity of the material with exposed (100) facet is lower than that of the (001)-facet-exposed materials, it is slightly higher than that of pristine TiO_2_, with values of 237.1, 215.8, 204.2, 189.5, 164.4, and 133.6 mAh g^−1^ at the corresponding current densities. The enhanced rate capability can be attributed to the increased specific surface area and a higher density of active sites on the exposed facets, facilitating faster ion diffusion and storage. Furthermore, the enhanced electronic conductivity of the exposed facets further promotes ion storage. The greater reversible discharge capacity of the (001)-facet-exposed materials further strengthens its rate performance. Figure S13a presents the galvanostatic charge–discharge curves of the (001)-facet-exposed materials at 1000 mA g^−1^, with an initial discharge capacity of 206.2 mAh g^−1^. The cycling performance of the three materials at a current density of 50 mA g^−1^ was also evaluated. As shown in Fig. 4c, after 180 cycles in the Mg–Li dual-salt electrolyte, the (001)-facet-exposed material retains a reversible capacity of 281.8 mAh g^−1^, significantly higher than that of the (100)-facet-exposed materials (154.62 mAh g^−1^) and pristine TiO_2_ (123.3 mAh g^−1^). Figure S13b presents the galvanostatic charge–discharge curves of the (001)-facet-exposed materials at 50 mA g^−1^, with an initial discharge capacity of 313.84 mAh g^−1^. Moreover, the (001)-facet-exposed material exhibits a coulombic efficiency close to 80% during cycling, indicating high reversibility and structural stability. Figure 4d presents the charge–discharge curves of the (001) facet at various current densities. As the current density increases, the specific capacity gradually decreases. Even at a high current density of 1000 mA g^−1^, the material maintains a well-defined discharge voltage plateau, demonstrating exceptional rate capability. To evaluate the stability of the cathode material in MLIBs, its cycling performance at 1000 mA g^−1^ was tested. As shown in Fig. 4e, the enhanced electronic conductivity of the (001)-facet-exposed material improves the ion storage capacity of the battery, contributing to its excellent stability. After 1070 cycles, the capacity remains at 170.6 mAh g^−1^, significantly higher than that of the (100)-facet-exposed materials (111.2 mAh g^−1^ after 749 cycles) and the pristine material (49.8 mAh g^−1^ after 364 cycles). And the TiO_2_ (001) electrode consistently maintains a high and stable coulombic efficiency during the long cycling process at 1000 mA g^−1^, further verifying its excellent electrochemical reversibility and cycling stability in Fig. 4e. Although at a lower current density (50 mA g^−1^), the TiO_2_ (001) electrode shows a coulombic efficiency slightly lower than 100%, which may be related to the slow kinetics of the Mg^2+^ de-intercalation process and the initial interface adjustment, its coulombic efficiency can remain stable under high current density. It indicates that the overall electrochemical reaction has good reversibility.

This advantage persists in a pure Mg salt electrolyte system, despite an overall decrease in capacity. The charge–discharge curves of the three materials in Mg salt electrolyte at current density of 50 mA g^−1^ are compared in Fig. S14a. The results indicate that the (001) facet material exhibits the highest discharge capacity (170.13 mAh g^−1^), surpassing the (100) facet (118.6 mAh g^−1^) and pristine anatase (89.61 mAh g^−1^), demonstrating a stronger Mg affinity of the (001) facet. Moreover, the charge–discharge profile of the (001) facet material with minimal polarization effects, indicating superior reaction kinetics and lower diffusion resistance. The (100) facet material exhibits slightly stronger polarization, while pristine anatase suffers from the most pronounced voltage hysteresis, which significantly hinders the intercalation and de-intercalation of Mg^2+^. The (001)-facet-exposed material continues to outperform the other two materials in rate capability tests at current densities ranging from 20 to 1000 mA g^−1^, delivering discharge capacities of 206.19, 172.89, 164.44, 148.9, 123.92, and 89.58 mAh g^−1^, respectively (Fig. S14b). Moreover, under prolonged cycling at 50 mA g^−1^, the capacity stabilizes at approximately 145.9 mAh g^−1^, with a coulombic efficiency of 82.6% (Fig. S14c). Figure S14d provides a detailed depiction of the charge–discharge profiles of the (001)-facet-exposed materials under different current densities in the MIBs system, demonstrating good rate adaptability. The capacity decline at high current densities is more pronounced, highlighting the relatively poor kinetic performance of the magnesium salt system. Figure S15a illustrates the galvanostatic charge–discharge curves at 500 mA g^−1^, where the initial discharge capacity is 134.7 mAh g^−1^. Figure S15b presents the galvanostatic charge–discharge curves of the (001)-facet-exposed materials at 50 mA g^−1^, with an initial discharge capacity of 185.64 mAh g^−1^. In long-term cycling tests at a high current density of 500 mA g^−1^ (Fig. S12e), the (001)-facet-exposed material retains a capacity of approximately 75.122 mAh g^−1^ after 1964 cycles, outperforming the (100)-facet-exposed materials (~ 57.23 mAh g^−1^ after 1233 cycles) and anatase (~ 33.7 mAh g^−1^ after 652 cycles).

To systematically investigate the impact of facet exposure on charge transport and ion diffusion, electrochemical impedance spectroscopy (EIS) was employed to evaluate the charge transfer resistance (*R*_ct_), while galvanostatic intermittent titration technique (GITT) was used to quantify the diffusion kinetics of Mg^2+^ and Li^+^ in the cathode materials [39]. The EIS profiles are fitted by the equivalent circuit (Fig. S16). As shown in Fig. 4f, in the Mg–Li dual-salt system, the Nyquist plots of the three materials exhibit a distinct semicircular shape, the (001)-facet-exposed material exhibits the lowest *R*_ct_, indicating superior charge transport capability. The (100)-facet-exposed TiO_2_ follows, while pristine anatase TiO_2_ exhibits the highest *R*_ct_, reflecting the poorest charge transfer kinetics. The specific *R*_ct_ values for the three materials after 20 cycles in the Mg–Li dual-salt electrolyte are shown in Table S1. To further verify ion diffusion capabilities in these materials, GITT was used to evaluate ion diffusion coefficients (D) during discharge. As depicted in Fig. 4g, h in MLIBs, the (001)-facet-exposed material exhibits a higher plateau voltage, longer discharge duration, and more stable voltage profiles, indicating superior ions storage performance and enhanced reaction reversibility. The ion diffusion coefficient of the (001) facet during intercalation ranges from 10^–11^ to 10^–7^ cm^2^ s^−1^, whereas pristine anatase TiO_2_ exhibits the lowest diffusion coefficient, ranging from 10^–14^ to 10^–9^ cm^2^ s^−1^. In the MIBs system, the overall impedance of all materials is higher than that of the MLIBs, with the (001) facet still demonstrating the most favorable interfacial charge transfer characteristics (Fig. S18a). The (100)-facet-exposed TiO_2_ follows, while pristine anatase TiO_2_ exhibits the highest *R*_ct_, reflecting the poorest charge transfer kinetics. The EIS profiles are fitted by the equivalent circuit (Fig. S17) and the specific *R*_ct_ values for the three materials after 20 cycles in the Mg electrolyte are shown in Table S2. GITT results further confirm that in the Mg salt system, the ion diffusion coefficient of the (001) facet during intercalation ranges from 10^–13^ to 10^–11^ cm^2^ s^−1^, significantly higher than that of the (100) facet and pristine anatase TiO_2_, the latter exhibiting the lowest diffusion coefficient, ranging from 10^–14^ to 10^–12^ cm^2^ s^−1^ (Fig. S18b, c). These findings confirm that the (001) facet exhibits superior charge transport properties in both electrolyte systems, with particularly pronounced performance enhancement in the Mg–Li dual-salt system.

To analyze the ion storage process in the electrode materials, cyclic voltammetry (CV) curves were collected in both Mg–Li and Mg electrolytes within a voltage range of 0.1–2.0 V at a scan rate of 0.01 mV s^−1^. As shown in Fig. S19, the CV curves of the Mg–Li system display well-defined redox peaks, suggesting that the storage process is dominated by ion intercalation/de-intercalation, consistent with a solid-state diffusion-controlled mechanism [16, 40, 41]. Comparing the CV curves of different materials, the (001)-facet-exposed TiO_2_ exhibits significantly higher peak currents than the (100)-facet-exposed and pristine anatase TiO_2_, highlighting the critical role of facet engineering in electrochemical performance. More importantly, as shown in Fig. S20, three materials exhibit distinct cyclic voltammetry (CV) responses in the Mg–Li dual-salt electrolyte, with facet-exposed TiO_2_ materials displaying notable differences in discharge voltage plateaus. Compared to pristine anatase TiO_2_, the (001) and (100) facet-exposed TiO_2_ not only exhibit higher discharge voltage plateaus, but also sharper, symmetric redox peaks with higher current values on the CV curves, reflecting stronger electrochemical reactivity and reversibility. This phenomenon is mainly attributed to the fact that the crystal surface modulation significantly reduces the band gaps of the TiO_2_ (001) and TiO_2_ (100) facets, which helps to enhance the electronic conductivity and interfacial charge transfer efficiency, thus accelerating the redox reaction kinetics. In addition, the adsorption energies of Mg^2+^ and Li^+^ on the surfaces of the two materials after exposure of the crystal faces are significantly better than those of intrinsic anatase and the ion diffusion energy barriers are also significantly reduced, which further promotes the migration and storage of ions, improves the reversible intercalation and de-intercalation of ions, and accelerates the electrochemical reaction kinetics of the materials, especially in the TiO_2_ (001) material. Additionally, nanostructuring, which reduces particle size, plays a crucial role in improving the discharge voltage plateau. In the Mg system, all three samples exhibit no distinct redox peaks (Fig. S21), indicating a typical pseudocapacitive behavior where Mg storage is primarily surface-controlled [42–44].

To further elucidate the Mg^2+^ storage mechanism, we quantitatively analyzed the diffusion-controlled and capacitive contributions to determine the underlying electrochemical reaction mechanisms. Figure 4i–k presents the CV curves of anatase TiO_2_, (001)-facet-exposed TiO_2_, and (100)-facet-exposed TiO_2_ electrodes at scan rates ranging from 0.1 to 1.0 mV s^−1^ in the MIBs [42, 43]. With increasing scan rates, all materials exhibit progressively larger current responses, and the CV curve shapes approach a rectangular profile, indicative of a dominant pseudocapacitive behavior [42]. Notably, the (001) facet-exposed TiO_2_ (Fig. 4j) displays significantly higher current responses at identical scan rates, implying superior electrochemical activity and enhanced capacitive performance. Quantitative analysis of diffusion- and capacitive-controlled contributions (Fig. 4l–n) reveals that capacitive contributions increase with scan rate for all materials. Specifically, for the (001)-facet-exposed TiO_2_, the capacitive contribution is 68% at 0.1 mV s^−1^ and increases markedly to 89% at 1.0 mV s^−1^, surpassing the (100)-facet-exposed TiO_2_ (85%) and pristine anatase TiO_2_ (74%). This result clearly demonstrates that the (001) facet provides more active sites, promoting a surface-controlled pseudocapacitive storage mechanism, making it a highly promising electrode material for MIBs. Additionally, to further reveal the energy storage mechanism, the CV curves of anatase TiO_2_, TiO_2_ (001), and (100) crystal facet materials at different scanning rates in the Mg–Li dual-salt electrolyte, and calculated the contribution ratios of the capacitance control and diffusion control processes as shown in Fig. S22a-c. The capacitive contributions of the three materials increase with the increase of the scanning rate, reflecting that the surface capacitive process gradually intensifies at high scanning rates. However, the overall calculation results show that the diffusion process still dominates. Among them, the diffusion proportion of TiO_2_ (001) crystal facet material exhibits the highest diffusion-controlled contribution, reaching 97.7% at 0.1 mV s^−1^ and remaining as high as 86.4% at 1.0 mV s^−1^, which was significantly higher than that of TiO_2_ (100) crystal plane (80.8%) and intrinsic anatase TiO_2_ (59.7%) in Fig. S22d-f. These results further confirm that the main energy storage mechanism in the dual-salt system is dominated by diffusion control. The intercalation pseudocapacitive nature of the ion storage mechanism indicates a significant enhancement in ion diffusion kinetics in MLIBs.

### Mechanistic Studies

**Fig. 5:**
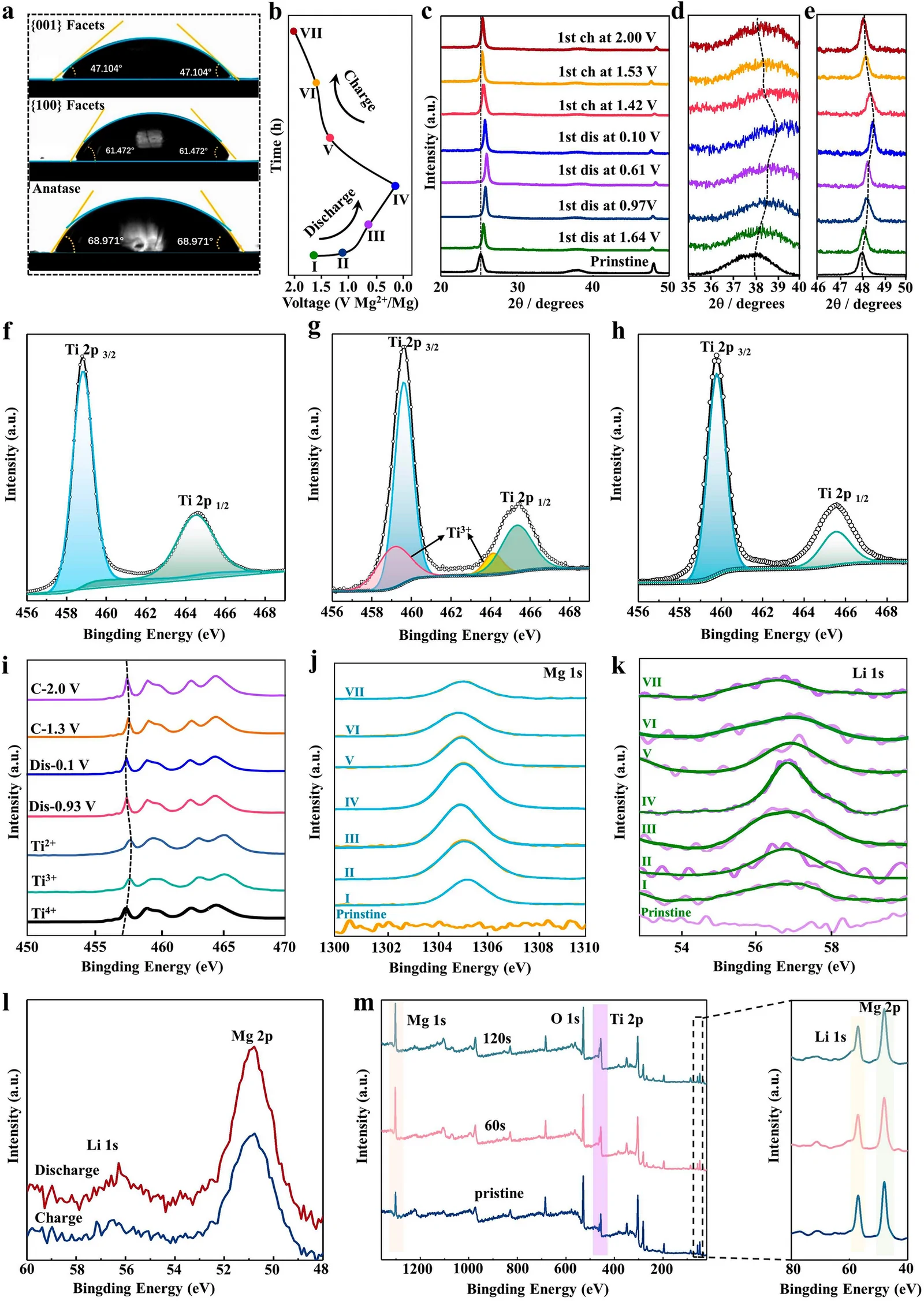
**a** Contact angles of APC + LiCl electrolytes with anatase TiO_2_, TiO_2_ (001) facet and (100) facet. **b** Voltage profiles of the TiO_2_ (001) facet cathode at different discharge and charge states corresponding to ex situ XRD tests. **c** Ex situ XRD patterns at different discharge and charge states. **d, e** Local enlarged images of XRD patterns at different states. **f**–**h** XPS spectra at high resolution of Ti 2*p* of TiO_2_ (001) facet materials. **i** Variation trend of different valence states of Ti element was measured by synchrotron radiation. **j, k** Ex situ XPS spectra of Mg 1*s*, and Li 1*s*.** l** XPS spectra of Li 1*s* and Mg 2*p* after 30 cycles discharge–charge processes at 50 mA g^−1^. **m** Depth-profiling XPS spectra of TiO_2_ (001) electrodes after 10 discharge cycles in Mg–Li dual-salt electrolyte

During charge–discharge cycles, electrode materials undergo periodic lattice expansion and contraction, and their structural stability directly impacts battery cycle life and energy storage performance. To investigate the structural evolution and electrochemical stability of TiO_2_ with different exposed facets in the Mg–Li dual-salt electrolyte system, this study systematically explores the effects of ion intercalation/de-intercalation on the crystal structure, Ti redox behavior, and ion storage mechanism using ex situ XRD, contact angle measurements, and XPS. As the concentration of charge carriers varies, the material undergoes expansion and contraction. The co-intercalation of Mg^2+^ and Li^+^ induces structural changes, as evidenced by the ex situ XRD patterns shown in Fig. S23a. After the first discharge, the most intense (200) diffraction peak shifts toward a higher angle, indicating a degree of lattice contraction upon ion intercalation. In contrast, when only Mg^2+^ intercalate into the (001)-facet-exposed materials in a pure Mg electrolyte (Fig. S23b), no significant peak shift is observed. This is attributed to the low Mg concentration in the crystal structure, which limits Mg^2+^ intercalation in the Mg system. Furthermore, as shown in Fig. S24, both the TiO_2_ (001) and (100) crystal facets materials exhibited shifts of the main diffraction peaks toward the high-angle direction during the first discharge process, reflecting a slight lattice contraction during the intercalation process. Among them, the peak position shifts of the (100) crystal facet material are more obvious than that of the pristine TiO_2_, indicating that its structural response is stronger during the Mg–Li co-intercalation process. In contrast, the peak position shift amplitude of the (001) crystal facet material is the largest and the ion co-intercalation is the most obvious. This further indicates that this crystal facet is conducive to promoting the kinetic process and electrochemical reversibility of the ion intercalation reaction. Notably, TiO_2_ (001) facet exhibits good structural reversibility in the Mg–Li system, as its diffraction peaks recover after charging. Beyond structural reversibility, no phase transition occurs during the intercalation/de-intercalation of TiO_2_ (001) facet, consistent with the modeled voltage profiles. The interfacial interaction between the electrode material and electrolyte plays a crucial role in charge transfer and ion diffusion kinetics. To further examine the electrochemical behavior and structural stability of the (001)-facet-exposed TiO_2_ in the Mg–Li dual-salt battery system, contact angle measurements (Fig. 5a) were conducted to compare the wettability of three different materials in Mg–Li dual-salt electrolytes. These measurements provide direct insight into the interaction between the electrode material and electrolyte. In the MLIBs, the contact angle of the (001)-facet-exposed materials is 47.10°. For the (100)-facet-exposed materials, the contact angles are 63.47°, pristine anatase TiO_2_ exhibits contact angles of 69.97° in the dual-salt electrolyte. These results indicate that facet exposure significantly enhances the affinity between the electrolyte and the electrode material, optimizing interfacial ion transport kinetics. Improved wettability not only reduces interfacial resistance but also minimizes side reactions during charge–discharge cycles, thereby enhancing rate performance and cycling stability. To further understand the Mg^2+^/Li^+^ storage behavior and the energy storage mechanism in the Mg–Li dual-salt electrolyte, ex situ XRD measurements were conducted to validate structural changes in the material. In Fig. 5b, the XRD patterns of the (001)-facet-exposed materials were recorded at different states within the voltage range of 0.1–2.0 V during the first cycle. As shown in Fig. 5c-e, the set of peaks is at ~ 25.2°, ~ 38.1°, ~ 48°, corresponding to the (101), (004), and (200) planes, respectively. During discharge, these peaks gradually shift toward higher 2θ angles, indicating the progressive intercalation of ions into the material, which induces internal lattice strain and leads to a degree of unit cell contraction. After charging to 2.0 V, the (101), (004), and (200) peaks gradually return to their initial positions, demonstrating the reversible extraction of intercalated Mg^2+^/Li^+^ and the structural stability of the material [43, 45, 46]. Throughout the charge–discharge process, no new diffraction peaks appear, nor do existing peaks disappear, confirming that the cathode material maintains a reversible phase transition during ion intercalation/(de)intercalation. By the end of the first cycle, these peaks nearly revert to their original positions, indicating that the structural integrity of the (001)-facet-exposed cathode remains intact after cycling, further verifying its excellent cycling stability.

To further elucidate the redox mechanism of the active Ti species in TiO_2_ (001) during electrochemical reactions, the valence state evolution of Ti at different voltage states were using XPS. Figure 5f presents the pristine Ti 2*p* spectrum of the (001) facet materials, where the binding energies of Ti 2*p*_3/2_ and Ti 2*p*_1/2_ are located at approximately 458.5 and 464.2 eV, respectively, indicating that Ti primarily exists in the Ti^4+^ oxidation state. Figure 5g displays the Ti 2*p* spectrum when the material is discharged to 0.1 V, where a characteristic peak of lower-valence titanium emerges at 456.5 eV, demonstrating the partial reduction of Ti^4+^ to Ti^3+^ [43, 47–49]. This indicates a significant decrease in the oxidation state of Ti. After charging to 2.0 V (Fig. 5h), the characteristic peaks corresponding to lower-valence titanium diminish or disappear, confirming that the reduced Ti species are re-oxidized to Ti^4+^. Synchrotron radiation XPS (SR-XPS) was employed to further investigate the valence state evolution of Ti (Fig. 5i). As the discharge voltage decreases progressively to 0.1 V, the valence state of Ti gradually reduces from Ti^4+^, with increasing signals of lower-valence Ti. Upon subsequent charging to 2.0 V, the lower-valence Ti species vanish, and the oxidation state of Ti returns to its initial Ti^4+^ state. These results confirm the highly reversible valence state transition of Ti during the co-intercalation and extraction of Mg^2+^ and Li^+^. To further investigate ion intercalation/(de)intercalation during charge–discharge cycles, XPS spectra were collected at different voltage states. The Mg 1* s* and Li 1* s* spectra at various stages are shown in Fig. 5j, k. Since residual Mg^2+^ and Li^+^ on the cathode surface are difficult to remove completely, their intercalation/(de)intercalation was analyzed based on the relative intensity of the peaks. During the discharge process (Stages I–IV), the characteristic peaks become progressively more pronounced, indicating a simultaneous magnesiation/lithiation process. During the charging process (Stages V–VII), these peaks gradually weaken and return to their original state, confirming that Mg^2+^ and Li^+^ are actively involved in the reaction and can be reversibly intercalated and extracted. These findings are consistent with the XRD analysis, further validating the reversibility of the reaction process. Figure 5l presents the XPS spectra of Li 1*s* and Mg 2*p* after 30 charge–discharge cycles at 50 mA g^−1^. The coexisting peaks of Li 1*s* and Mg 2*p* at 55.5 and 50.5 eV, respectively, provide additional evidence that Mg^2+^ and Li^+^ can simultaneously intercalate and de-intercalate from the host material in a reversible manner [42]. In addition, by comparing the GCD curves of TiO_2_ (001) facet material under three different electrolyte systems (pure Mg salt, pure Li salt, and Mg–Li dual-salt), it can be found that TiO_2_ (001) exhibits the highest and most stable discharge voltage plateau in the Mg–Li dual-salt system. The charge and discharge behaviors that are significantly different from the other two single-ion electrolyte systems are shown in Fig. S25. This feature further verifies the co-intercalation of Mg^2+^ and Li^+^ in the dual-salt electrolyte. Meanwhile, we verified the distribution of ions inside the electrode through XPS tests at different sputtering depths (Fig. 5m). The test results show that with the increase of sputtering depth from no etching to 120 s of etching, obvious Mg and Li signals can still be detected, confirming that the Mg and Li are not only on the surface but have undergone deep intercalation. Therefore, all the above results fully confirm that existence of Mg–Li co-intercalation of TiO_2_ (001) in the dual-salt electrolyte. To investigate the structural stability after Mg–Li co-intercalation, TEM and EDS elemental mapping analyses were conducted on samples after 30 charge–discharge cycles. As shown in Fig. S26a, b, TEM results reveal that the sample retains its intact sheet-like structure even after 30 cycles, demonstrating excellent structural stability during cycling without significant particle fragmentation. Elemental mapping confirms the uniform distribution of Ti and O in both the discharged and charged states. To further evaluate the structural stability and integrity of the material after prolonged cycling, TEM and EDS elemental mapping analyses were performed on samples after 350 cycles (Fig. S27). TEM images show that the material maintains its sheet-like morphology without any noticeable structural damage. The EDS elemental mapping provides direct evidence of compositional stability, as Ti and O remain uniformly distributed within the sheet-like structure. This demonstrates that the material exhibits outstanding stability over long-term cycling, which is crucial for maintaining sustained electrochemical performance. The material maintains structural stability during Mg–Li co-intercalation, which plays a crucial role in enhancing capacity, improving cycling stability, and optimizing kinetic properties. These studies further validate that facet engineering, combined with Mg–Li co-intercalation, significantly enhances the electrochemical stability and reversibility of TiO_2_ in energy storage systems, providing theoretical guidance and experimental support for the design of high-performance Mg-based battery electrode materials.

The stability of the anode interface is critical for the long-term cycling performance of MIBs. To evaluate the morphological evolution and chemical stability of the Mg anode after prolonged cycling, SEM characterization was performed. The surface remained dense and free of dendrites (Fig. S28a), indicating that the electrodeposition process resembles that of metallic Mg rather than Li. EDS elemental mapping (Fig. S28b, c) and XPS analysis of the electrode surface confirmed the exclusive presence of Mg (Fig. S28d, e), demonstrating the absence of Li co-deposition in this system. Given that the electrodeposition voltage is − 0.1 V and Mg is positioned at 0.6 V above Li^+^/Li^0^, this behavior is expected [50]. Nevertheless, our findings highlight the primary advantages of using a Mg anode, including dendrite-free deposition and quasi-stability.

### Pouch Cells

**Fig. 6:**
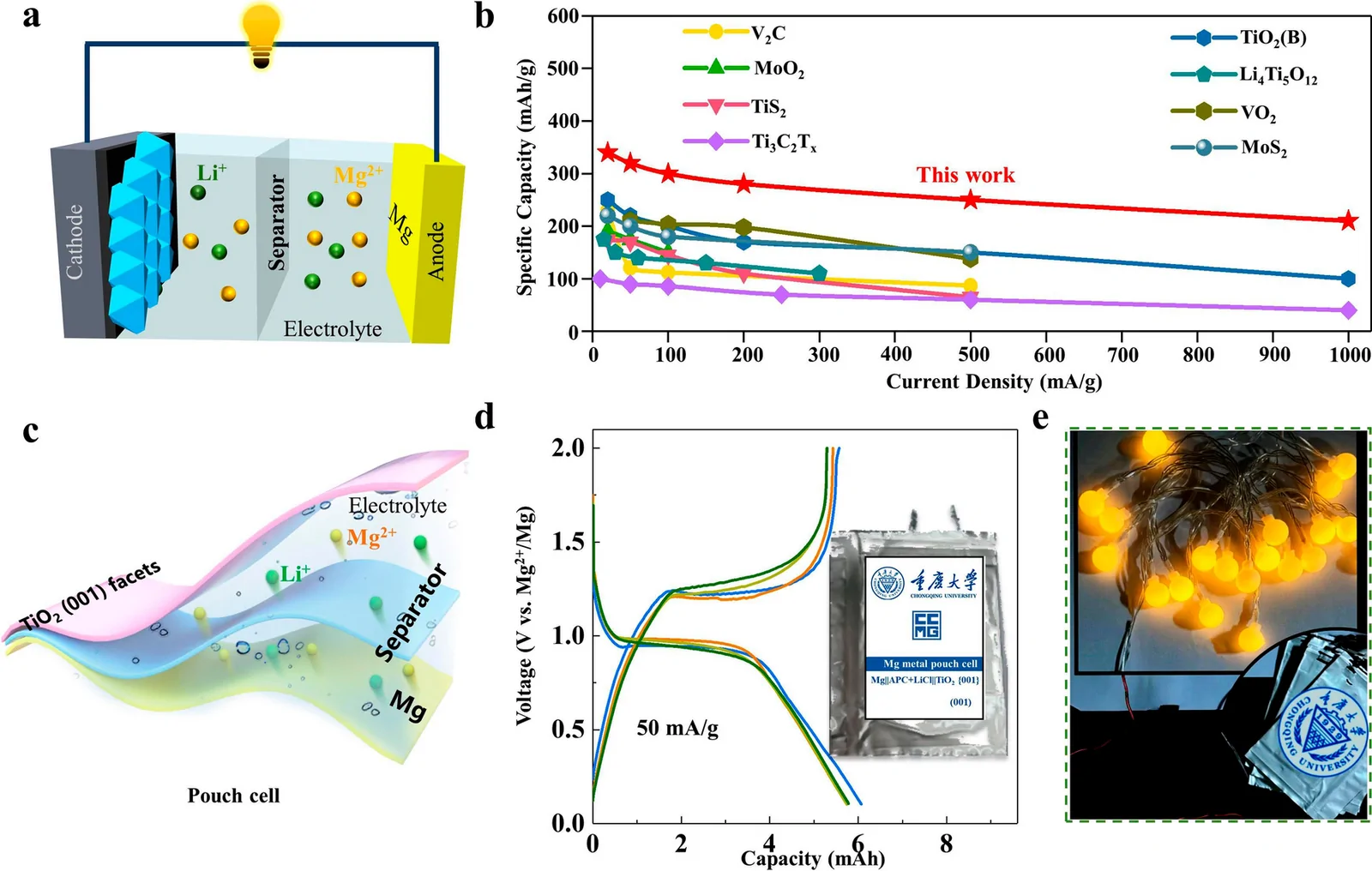
**a** Schematic illustration of the working principle in full-cell device. **b** Comparison of the electrochemical performance with other reported materials. **c** Schematic diagram of pouch-cell device. **d** Charge/discharge profiles of the Mg||APC + LiCl|| TiO_2_ (001) facet pouch cell at 50 mA g^−1^. **e** 20 LEDs lightened by two Mg|| APC + LiCl|| TiO_2_ (001) facet pouch cells connected in series

Figure 6a presents a schematic illustration of the full cell, comprising a cathode and a Mg metal anode, where the cathode is TiO_2_-based material. The electrolyte consists of APC and APC + LiCl. As shown in Fig. 6b, TiO_2_ (001) exhibits superior rate capability and stability in Mg–Li hybrid batteries compared to other state-of-the-art cathode materials, such as VO_2_, MoO_2_, MoS_2_, TiS_2_, spinel Li_4_Ti_5_O_12_, Ti_3_C_2_T_x_ MXene, V_2_C MXene, and TiO_2_(B) [16, 42, 51–54]. In addition, Fig. S29 highlights the exceptional cyclic stability and the admirable reversible capacity of this work in comparison with other reported Mg–Li hybrid batteries. Figure 6d depicts the typical charge–discharge profiles of TiO_2_ (001) facet in Mg–Li dual-salt and Mg salt electrolytes at a current density of 50 mA g^−1^. In the Mg–Li dual-salt electrolyte, two distinct voltage plateaus are maintained. The total discharge capacity of the Mg||APC + LiCl|TiO_2_ (001) facet pouch cell reaches up a ~ 6 mAh. Additionally, a series connection of three Mg||APC + LiCl|TiO_2_ (001) facet pouch cells successfully powered 20 yellow LEDs (operating voltage: 1.8–2.4 V, Fig. 6e). Furthermore, the pouch-cell performance in a pure Mg salt electrolyte is shown in Fig. S30a, where a discharge capacity of 2 mAh is achieved, and it can also stably power LEDs continuously. Figure S30b, c, respectively, shows the GCD curves of TiO_2_ (001) pouch cells during charging and discharging at a current density of 50 mA g^−1^ under the conditions of Mg–Li dual-salt electrolyte and pure Mg salt electrolyte. It can be seen that the battery exhibits a higher and more stable voltage level in the dual-salt system. Furthermore, as shown in Fig. S30d, the TiO_2_ (001) electrode still maintains a capacity retention rate of 77.11% after 378 cycles in the Mg–Li dual-salt electrolyte, and can achieve a capacity retention rate of 46.37% after 204 long cycles in the pure Mg electrolyte system. These results convincingly demonstrate the excellent compatibility between the TiO_2_ (001) facet cathode and the ultrathin Mg metal anode (0.2 mm), enabling stable electrochemical performance. This system holds significant promise for applications in flexible energy storage devices, wearable electronics, and low-power applications.

## Conclusion

In summary, anatase TiO_2_ nanostructures with exposed (001) and (100) facets were successfully synthesized via a hydrothermal method, employing a strategy of nanostructural tuning to expose specific crystal facets. The energy storage mechanisms and kinetic properties of these materials were systematically investigated. Theoretical calculations show that the exposure of high-energy facets optimizes the material electronic structure, reduces the bandgap, and enhances electronic conductivity. Furthermore, calculations indicate that nanostructural engineering with specific facet exposure significantly reduces the diffusion energy barriers for Mg^2+^ and Li^+^. Notably, on the (001) facet, the diffusion barriers for Mg^2+^ and Li^+^ were reduced to 0.475 and 0.381 eV, respectively, with ion diffusion coefficients improving by 2–3 orders of magnitude compared to conventional TiO_2_ (Mg^2+^: from 10^–13^ to 10^–11^ cm^2^ s^−1^; Li^+^: from 10^–11^ to 10^–7^ cm^2^ s^−1^). This optimization significantly facilitates ion insertion/extraction, thereby improving rate capability and cycling stability. Additionally, DFT calculations reveal significant differences in Mg^2+^ adsorption on different facets. Both (001) and (100) facets exhibit relatively negative ion adsorption energies, with the adsorption energy for Mg^2+^ being lower than that for Li^+^, and the (001) facet showing a stronger affinity for Mg^2+^. This stronger interaction promotes Mg^2+^ interfacial storage. Experimental results demonstrate that TiO_2_ nanocrystals with exposed (001) facet show a higher capacity increase in pure Mg salt electrolytes (90.24%) compared to Li salt electrolytes (70.77%). Moreover, the capacity increase in Mg–Li dual-salt electrolytes is higher than that in the Li salt system. These results indicate that the interfacial Li storage effect is limited, and controlling the nanocrystal facets effectively enhances multivalent ion storage. The (001) facet exhibits outstanding performance in MLIBs, maintaining a reversible capacity of 170.6 mAh g^−1^ after 1070 cycles at 1000 mA g^−1^. In MIBs, (001) facet retains approximately 75 mAh g^−1^ after 1964 cycles at 500 mA g^−1^. Furthermore, compared to the bulk TiO_2_, the (100) facet also demonstrates superior electrochemical performance in Mg–Li dual-salt electrolytes (111.2 mAh g^−1^ after 749 cycles at 1000 mA g^−1^) and pure Mg salt electrolytes (57.23 mAh g^−1^ after 1233 cycles at 500 mA g^−1^). This study shows that the rate performance and specific capacity of the electrode materials can be significantly improved by nanocrystalline crystal facets control.

## Supplementary Information

Below is the link to the electronic supplementary material.

Supplementary file1 (DOCX 11200 kb)
