# Supplementary material for: Nanosized Anatase TiO2 with Exposed (001) Facet for High-Capacity Mg2+ Ion Storage in Magnesium Ion Batteries
Source: Nanomicro Lett. 2025 Aug 1;18:17. doi: 10.1007/s40820-025-01861-7 (PMC12316663; doi:10.1007/s40820-025-01861-7)
Supplement: Supplementary file 1 — Supplementary file1 (DOCX 11200 kb) [file 40820_2025_1861_MOESM1_ESM.docx]

**Nanosized Anatase TiO_2_ with Exposed (001) Facet for High-Capacity Mg^2+^ Ion Storage in Magnesium Ion Batteries**

Rong Li^1,2^, Liuyan Xia^1,2^, Jili Yue^1,2,^*, Junhan Wu^1,2^, Xuxi Teng^1,2^, Jun Chen^3^, Guangsheng Huang^1,2,^*, Jingfeng Wang ^1,2^, Fusheng Pan^1,2,^*

^1^ National Engineering Research Center for Magnesium Alloys, National Innovation Center for Industry-Education Integration of Energy Storage Technology, College of Materials Science and Engineering, Chongqing University, Chongqing 400044, P. R. China

^2^ Chongqing Institute of New Energy Storage Materials and Equipment, Chongqing 401135, P. R. China

^3^ Department of Materials, University of Oxford, Oxford, OX1 3PH, United Kingdom

*Corresponding authors. E-mail: [jili.yue@cqu.edu.cn](mailto:jili.yue@cqu.edu.cn) (Jili Yue); [gshuang@cqu.edu.cn](mailto:gshuang@cqu.edu.cn) (Guangsheng Huang); [fspan@cqu.edu.cn](mailto:fspan@cqu.edu.cn) (Fusheng Pan)

**S1 Electrochemical analytical method**

The galvanostatic intermittent titration technique (GITT) is performed at 50 mA/g for 5 min, followed by a relaxation period of 30 min. The value of the D_Mg_^2+^ can be obtained based on the simplified Fick's second law by the following equation [S1, S2]:

$$\begin{aligned} D = \frac{4}{\left( \pi\tau\right)}\left( \frac{m_{B}V_{M}}{M_{B}S} \right)\left( \frac{\Delta E_{s}}{\Delta E_{t}} \right) \#(S1 \end{aligned})$$

where τ is the time of current pulse. V_M_, m_B_, and M_B_ represent molar volume, the mass, and molar mass of materials, respectively. S is the contact area of electrode/electrolyte. $\Delta E_{s}$and $\Delta E_{t}$ are the steady-state potential change by the current pulse and potential change during the constant current.

The b-values of the contribution of capacitance-controlled and diffusion capacities can be obtained according to Eqs. S2 and S3. The quantifiable proportion of the pseudocapacitive/diffusion control contribution is based on Eq. S4 [S3].

$$\begin{aligned} i=av^{b}\#(S \end{aligned}2)$$

where 𝑎 and 𝑏 both are constants, 𝑖 is the peak current value (A), 𝑣 is the different scan rate (mV/s).

The electrochemical reaction type can also be summarized via the relationship among scan rate (v) and peak current (i) in CV curves under different scan rates, which follows the equation of [S1, S3, S4]:

$$\begin{aligned} \log\left( i \right)= b\log\left( v \right)+\log\left( a \right)\#(S3) \end{aligned}$$

where the b value is used to distinguish electrochemical reaction behaviors. When the b value is close to 0.5, electrochemical reactions are dominated by diffusion-controlled process; when the b value is approaching to 1.0, capacitive process become the dominant factor in electrochemical reactions.

To further confirm the capacitive contribution of the current response (i) at a certain voltage (V), the corresponding pseudocapacitance and diffusion-controlled behaviors in the CV results were measured and calculated by the following Eq. S2 [S1, S4]:

*i*(V) = *k₁v* + *k₂v*¹ᐟ² (S4)

where 𝑖 is the peak current value (A), 𝑣 is the different scan rate (mV/s), V is the specified voltage (V), and 𝑘_1_ and 𝑘_2_ are adjustable parameters.

**S2 Supplementary Figures and Tables**


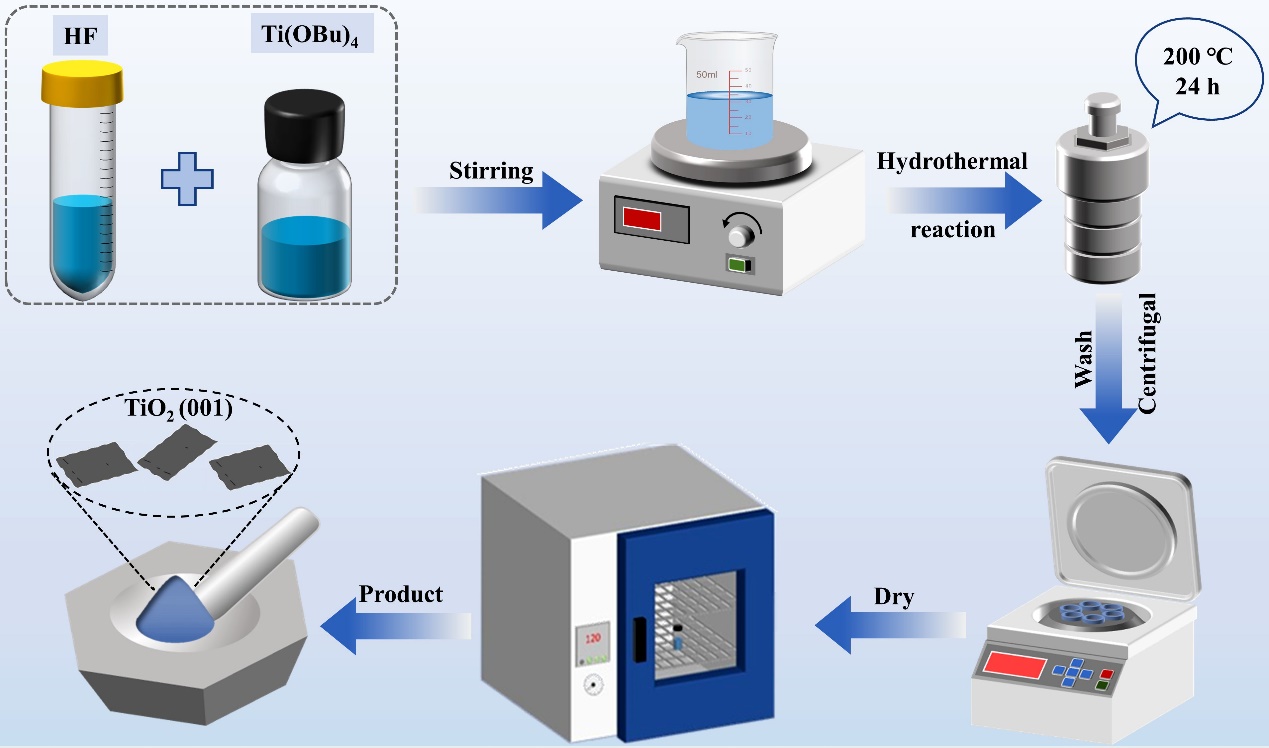


**Fig. S1** Schematic illustration of the synthesis process of TiO_2_ (001) facet materials


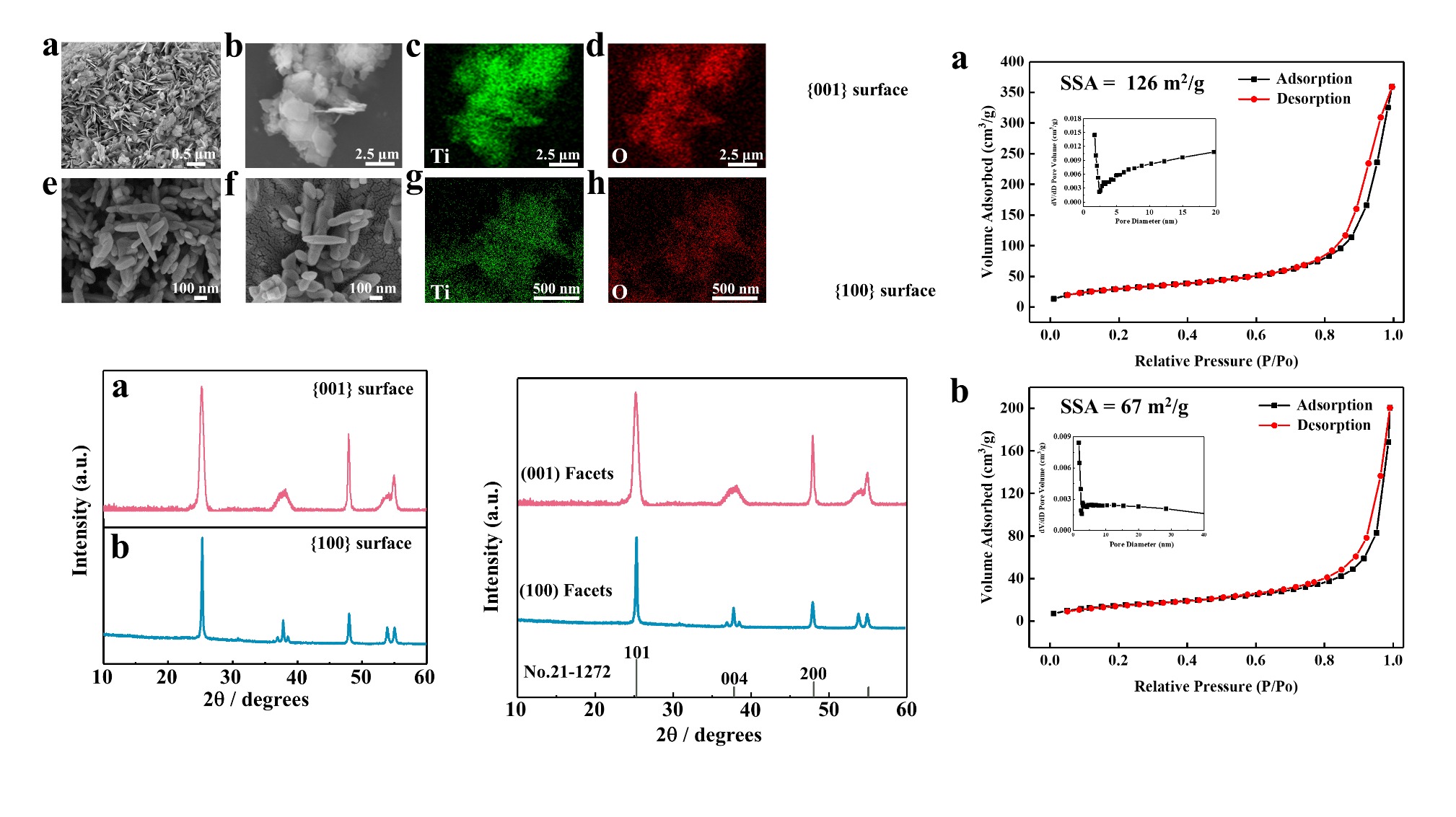


**Fig. S2** XRD patterns of TiO_2_ (001) facets and TiO_2_ (100) facets


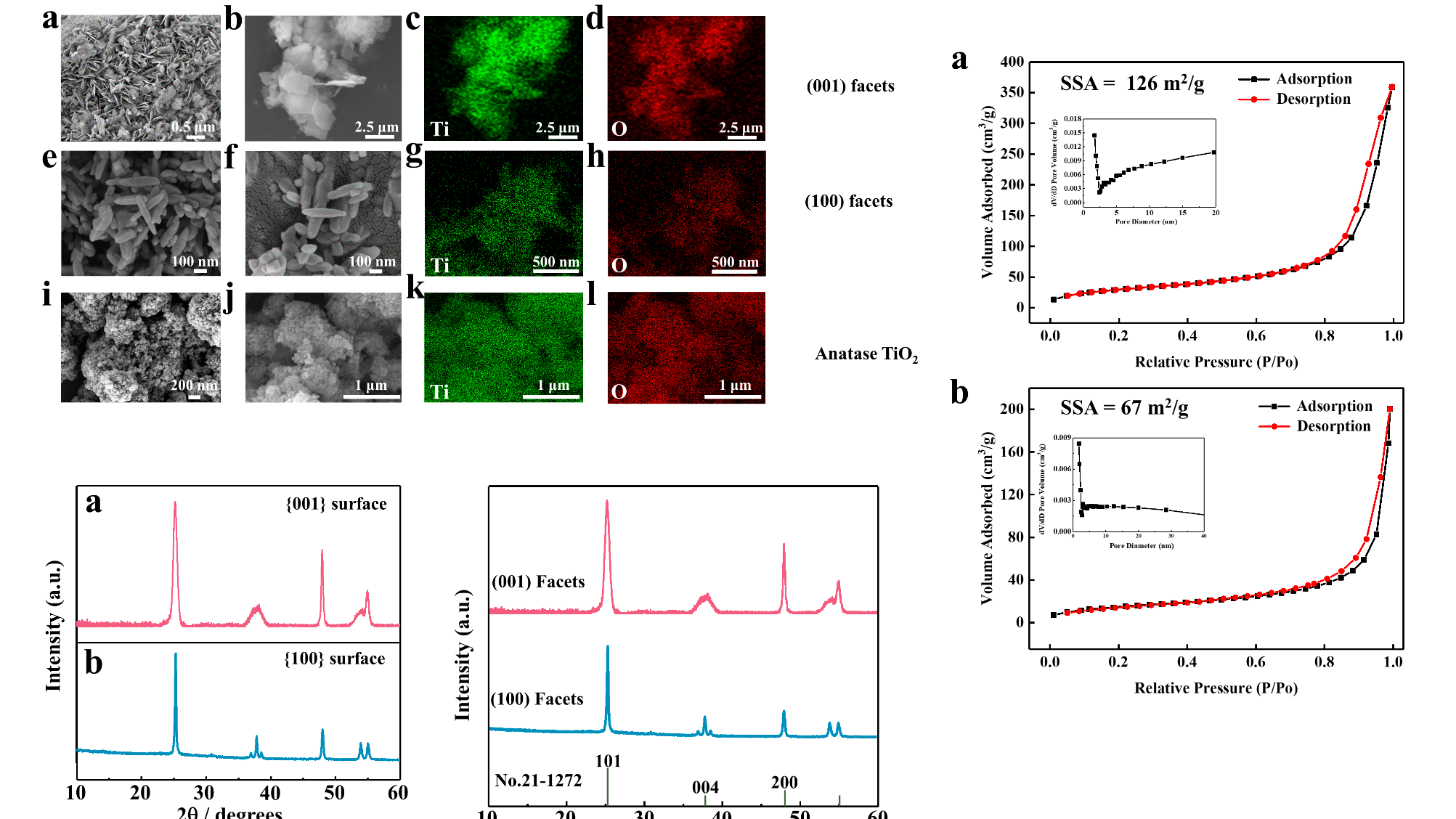


**Fig. S3** SEM and Elemental mapping images of **a-d** TiO_2_ (001) facets, **e-h** TiO_2_ (100) facets and **i-l** anatase TiO_2_


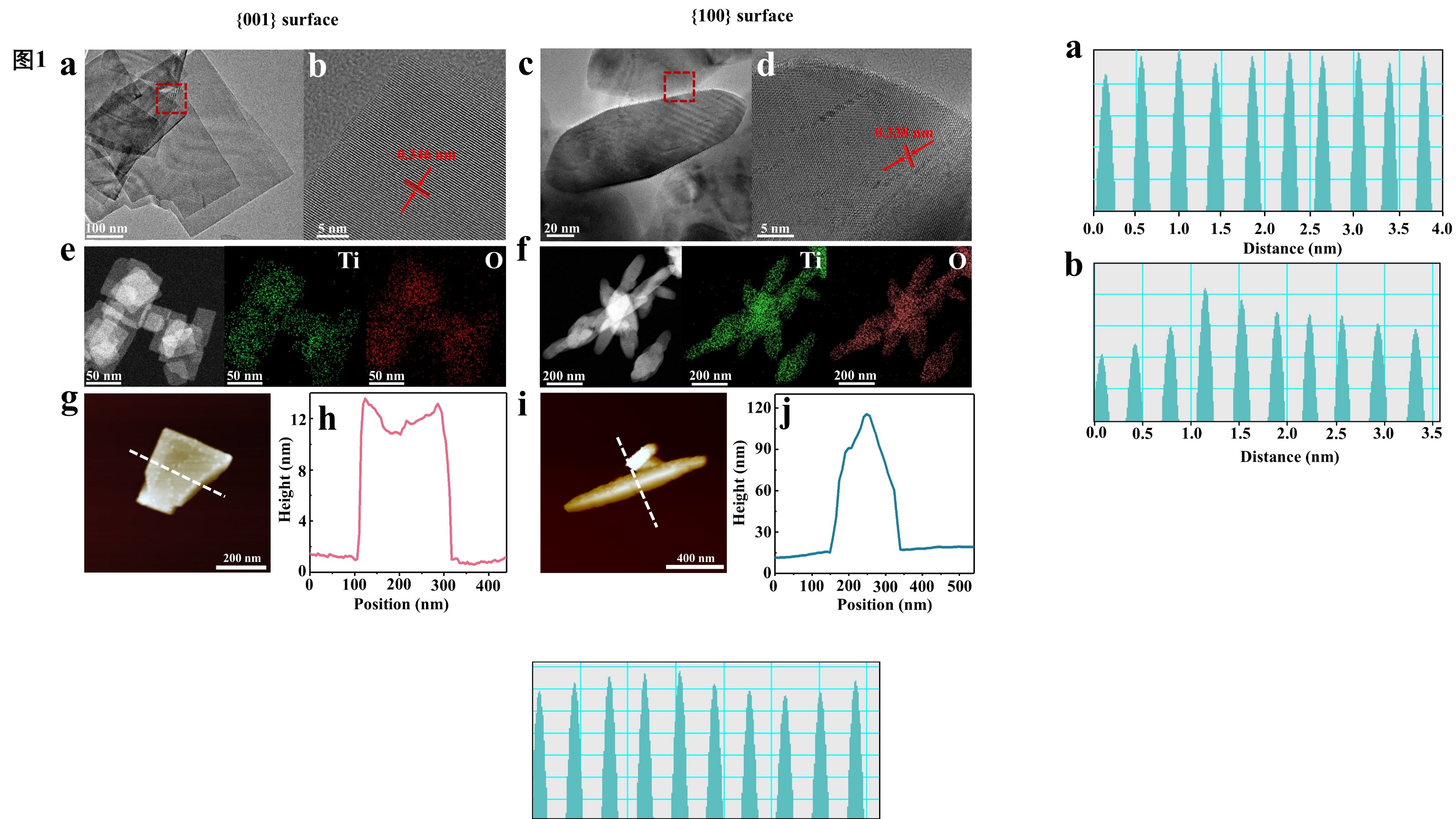


**Fig. S4** Brightness profiles along the dotted lines in **a** TiO_2_ (001) facets and **b** TiO_2_ (100) facets


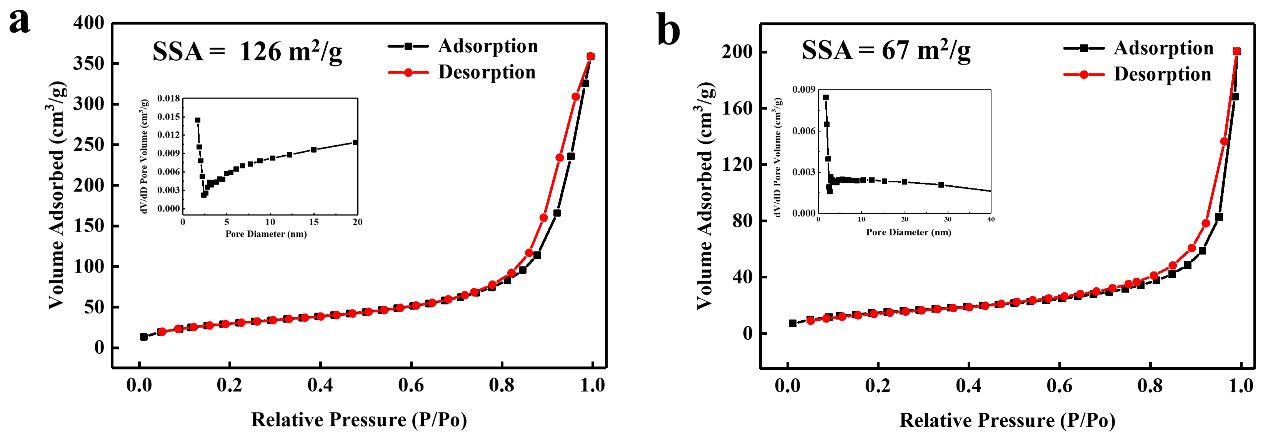


**Fig. S5** N_2_ adsorption-desorption isotherm curves and Pore-size distribution curves of **a** TiO_2_ (001) facets and **b** TiO_2_ (100) facets


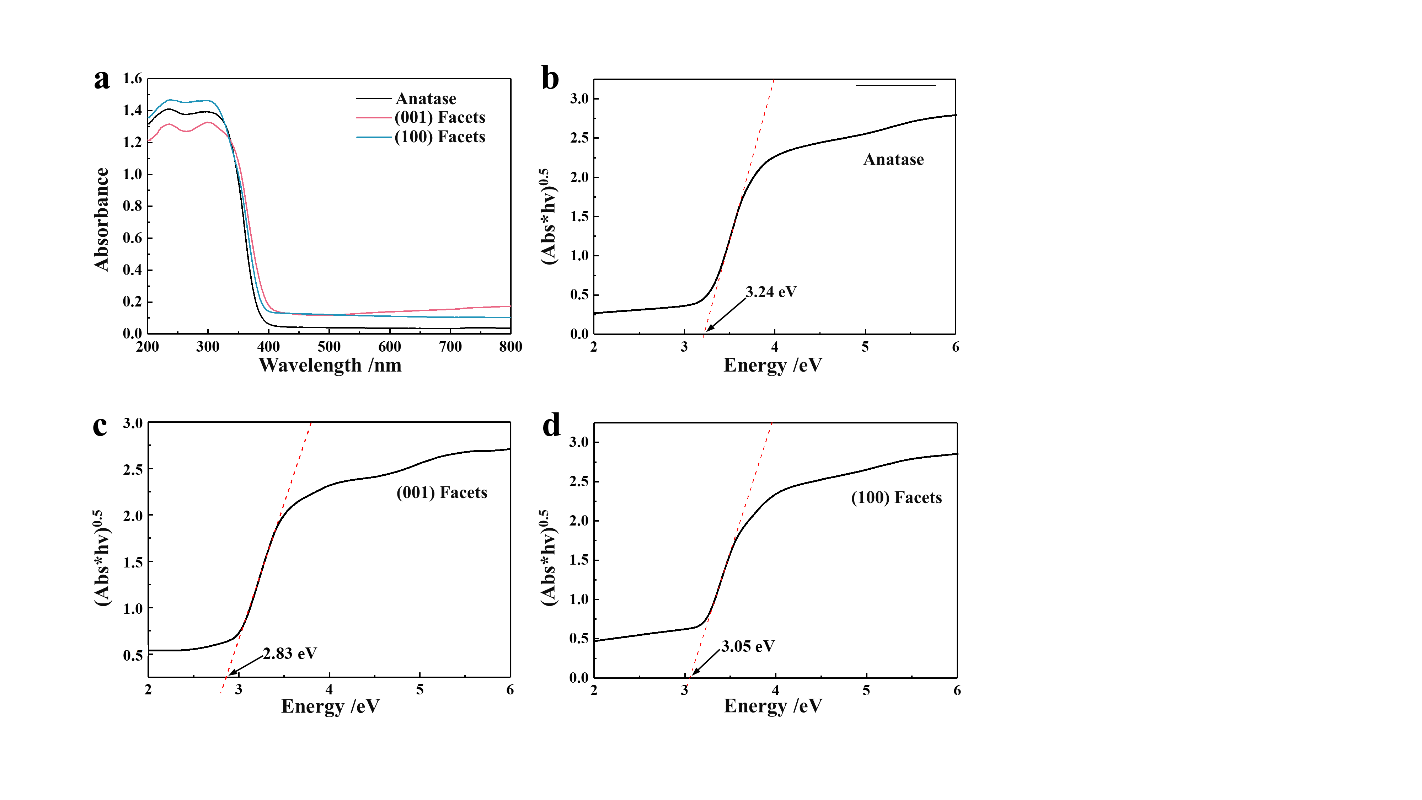


**Fig. S6 a** The UV−visible absorption spectrum of the TiO_2_ samples. **b-d** Their corresponding plots of transformed Kubelka−Munk function versus the energy of photon


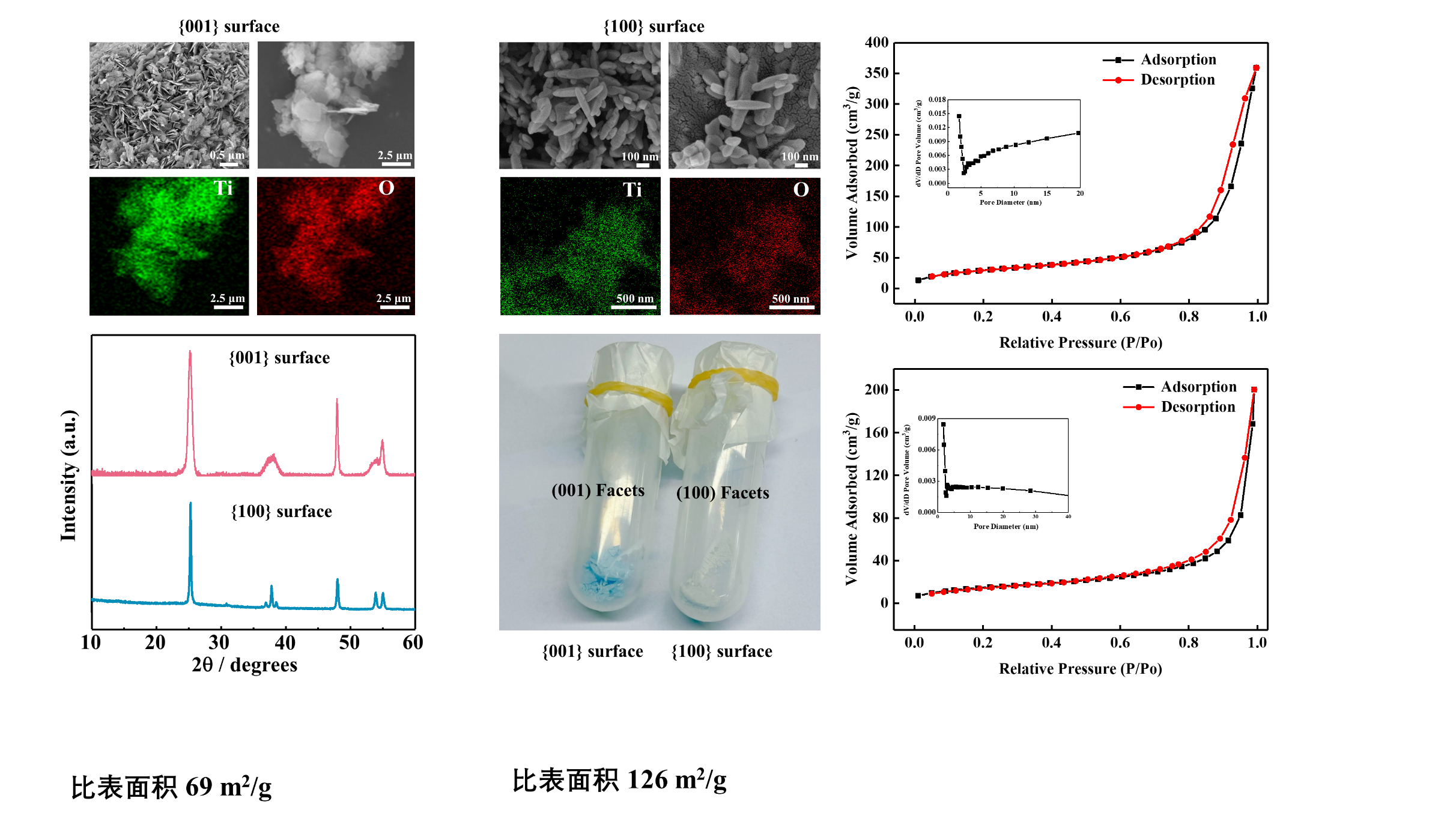


**Fig. S7** Color comparison of the synthesized sample TiO_2_ (001) facets (left) and TiO_2_ (100) facets (right)


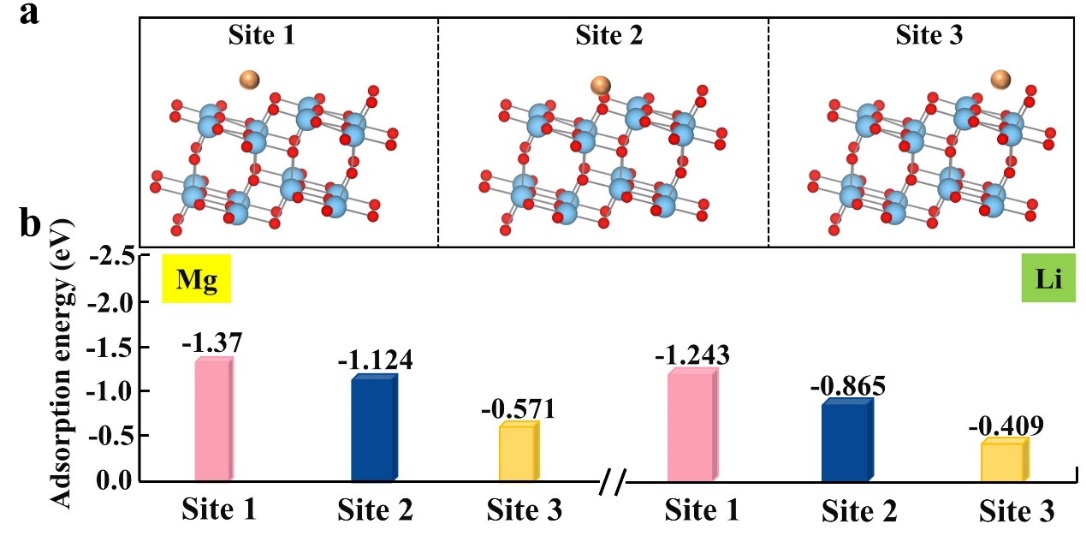


**Fig. S8** **a** Schematic illustration of Mg^2+^ and Li^+^ adsorption sites on the TiO_2_ (101) facet, with different adsorption sites labeled as Site 1, Site 2, and Site 3. **b** Adsorption energies of Mg^2+^ and Li^+^ at different adsorption sites on the TiO_2_ (101) facet


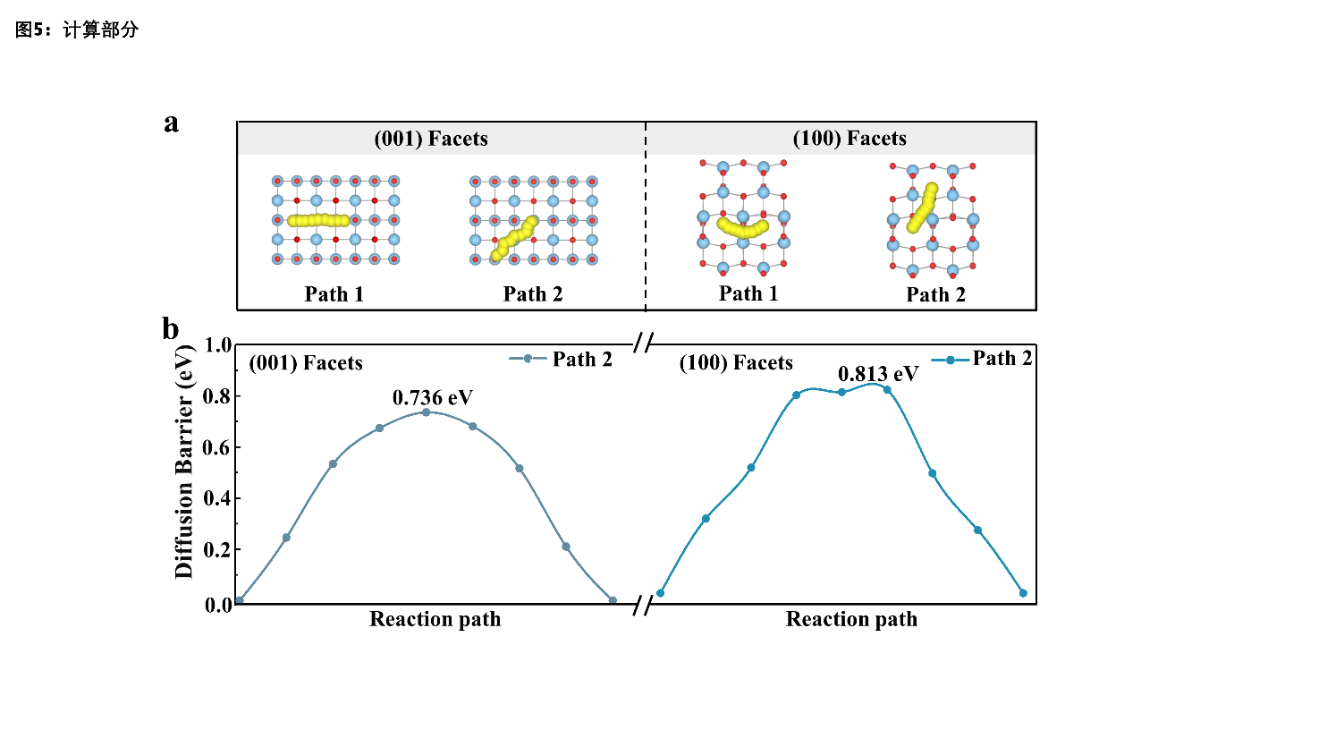


**Fig. S9** **a** Diffusion energy barrier and **b** diffusion paths the Mg^2+^ at TiO_2_ (001) facets and (100) facets


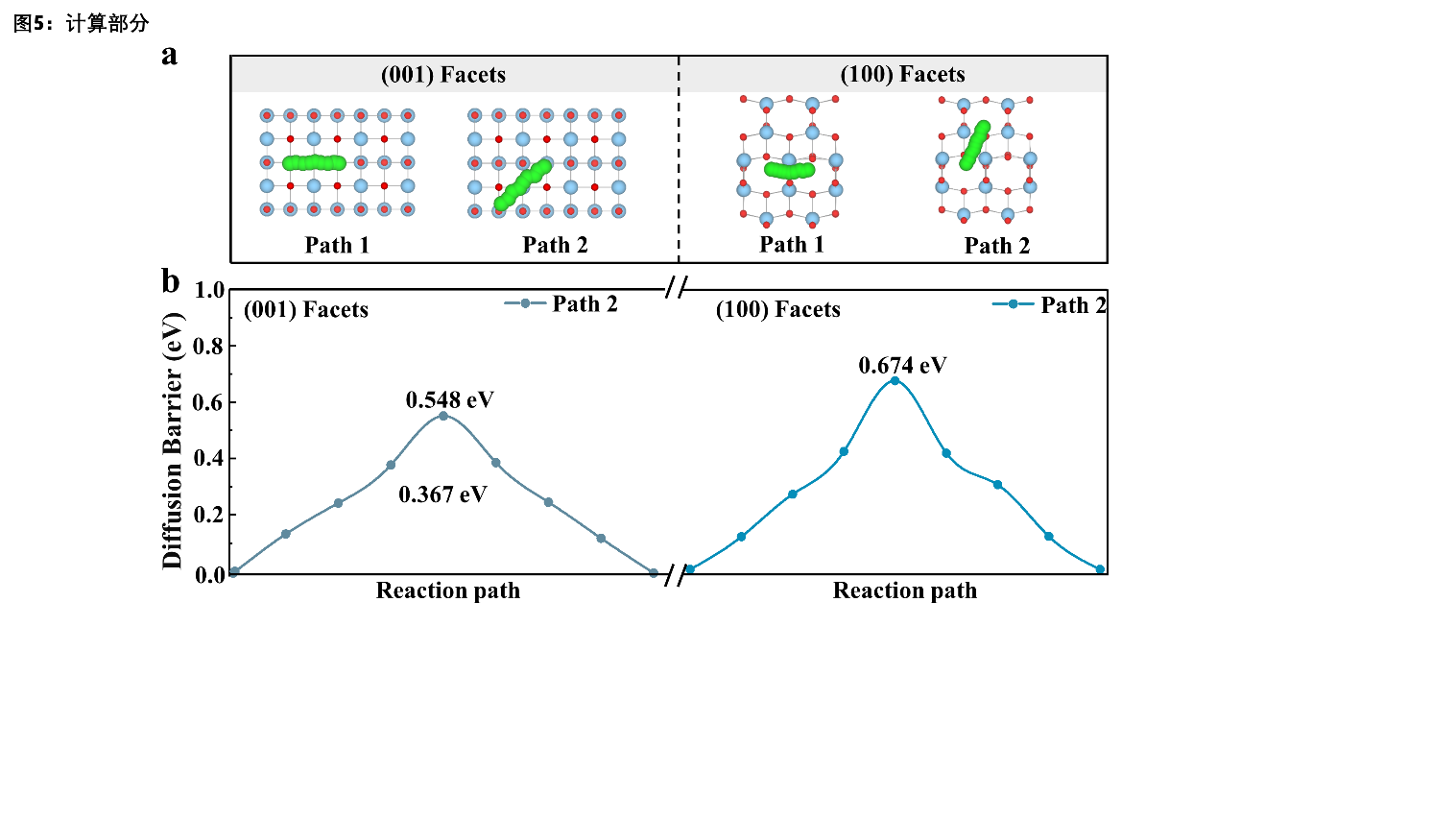


**Fig. S10** **a** Diffusion energy barrier and **b** diffusion paths the Li^+^ at TiO_2_ (001) facets and (100) facets


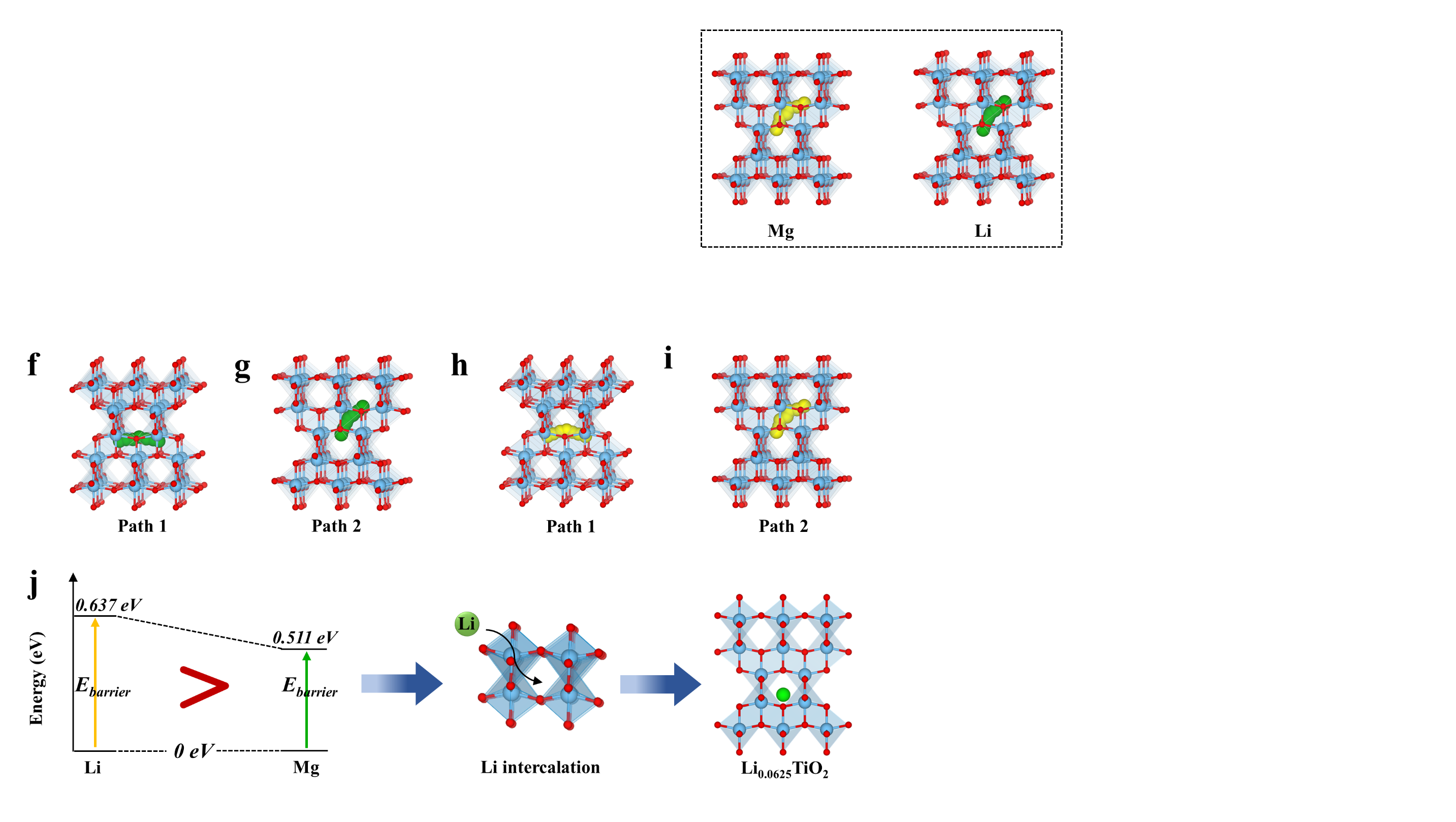


**Fig. S11** Diffusion paths the Mg^2+^ and Li^+^ at TiO_2_ (001) facets and (100) facets in anatase TiO_2_


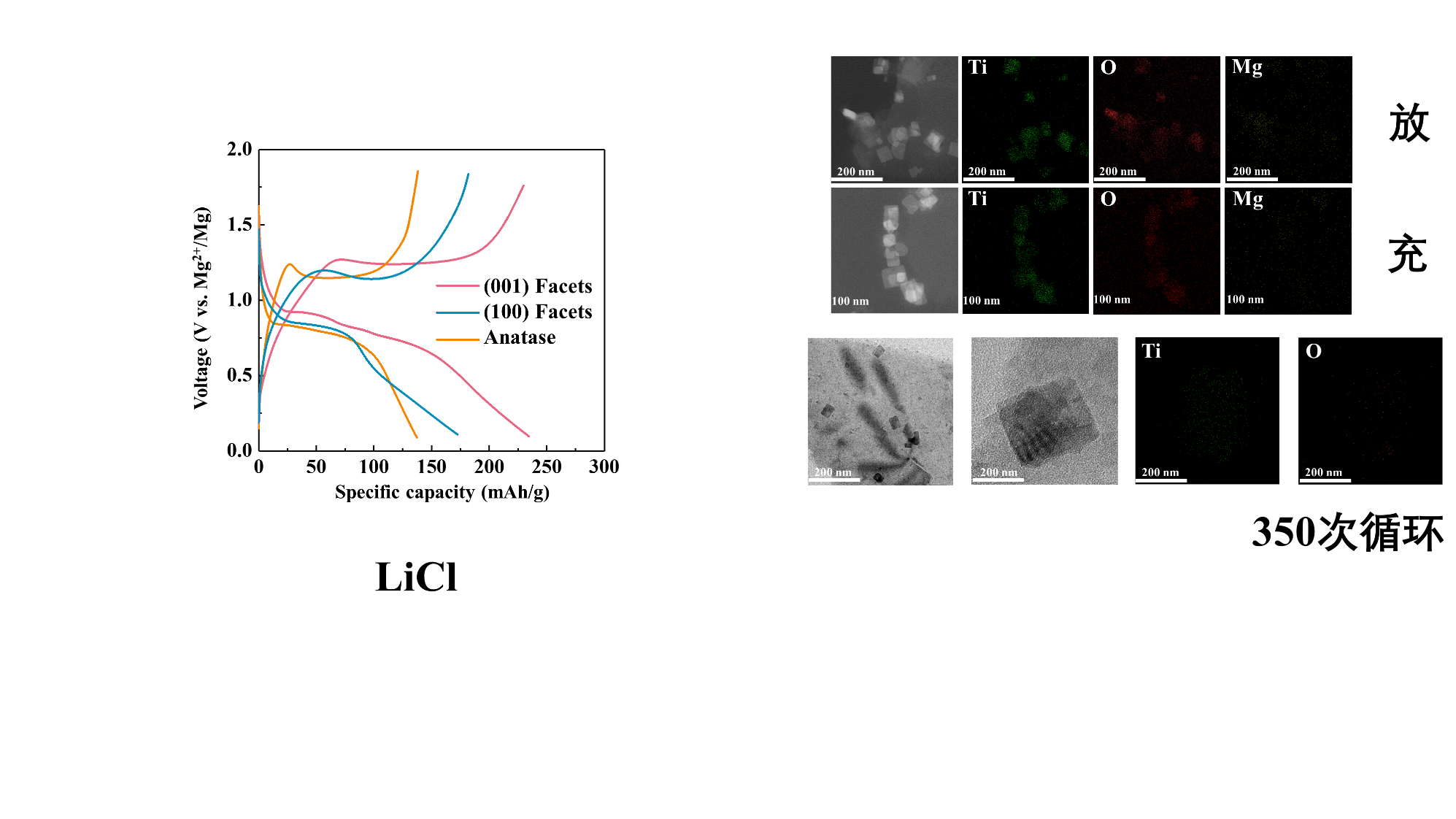


**Fig. S12** Voltage profiles of of TiO_2_ (001) facets, TiO_2_ (100) facets and anatase TiO_2_ at 50 mA/g in Li salt electrolyte


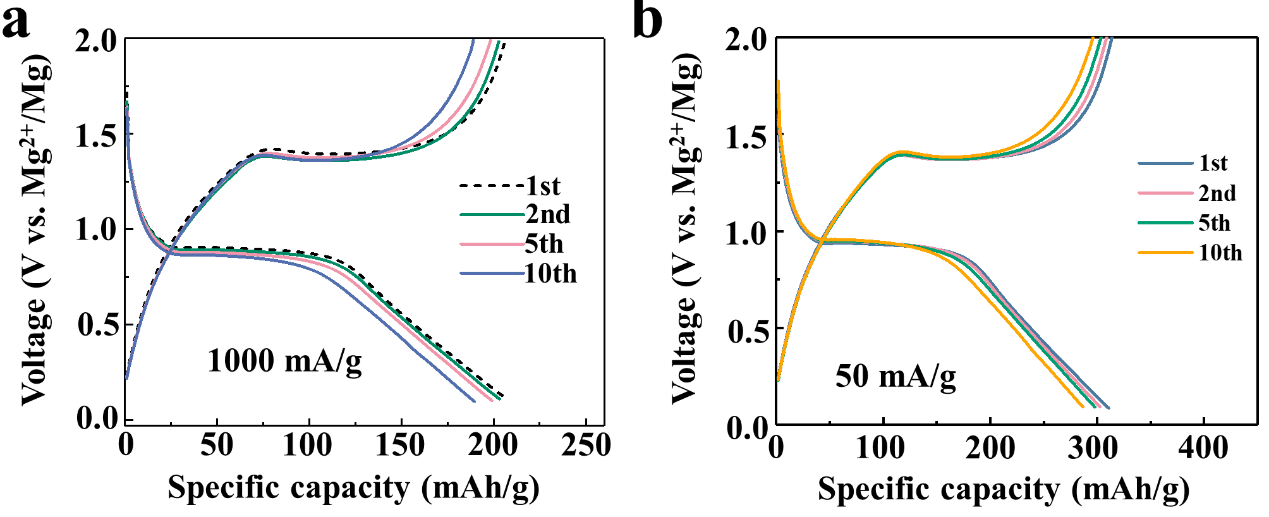


**Fig. S13** **a** Voltage profiles of the TiO_2_ (001) facets in MLIBs at 50 mA/g and b 1000 mA/g


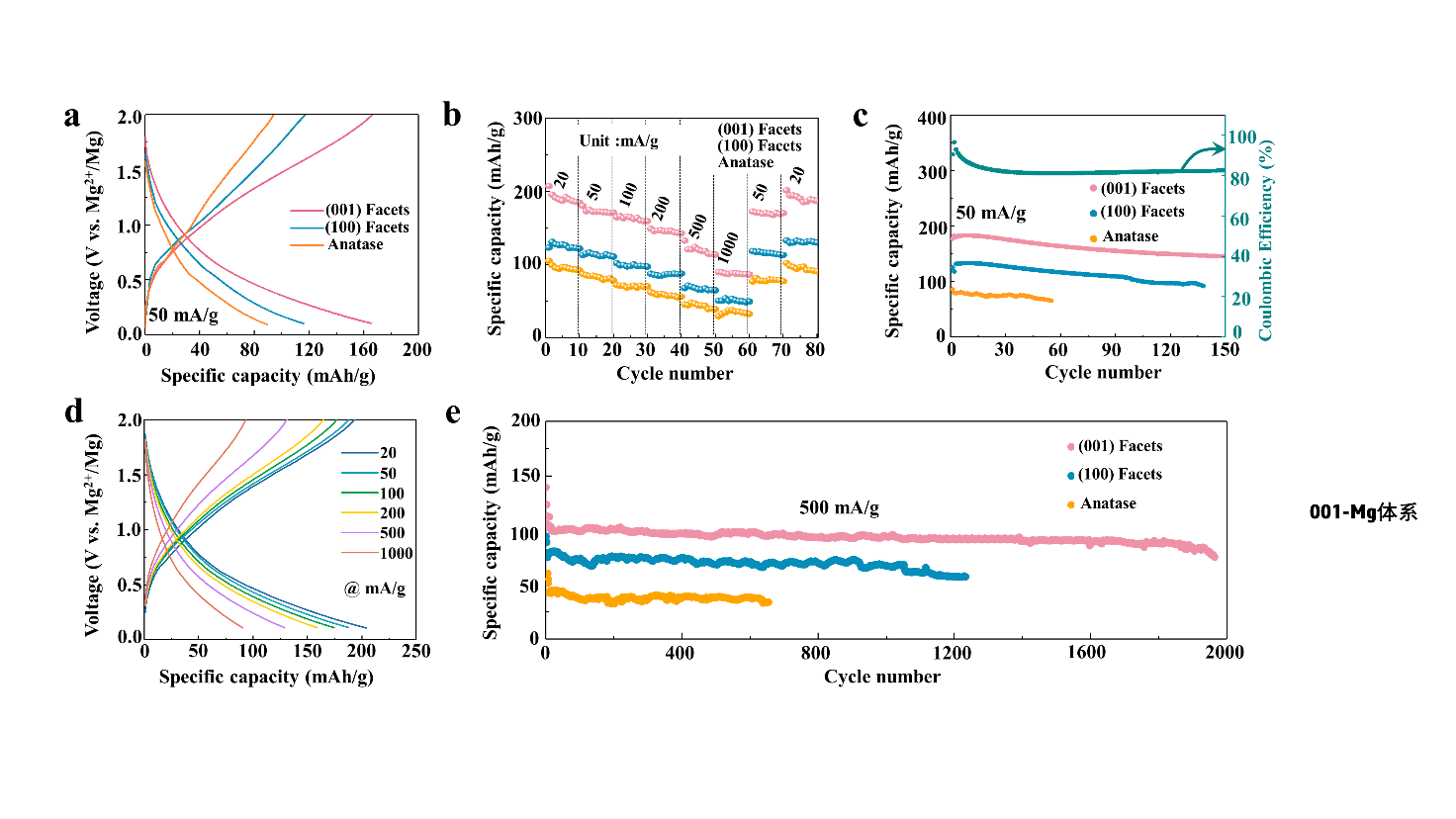


**Fig. S14 a** Charge-discharge curves of anatase TiO_2_, TiO_2_ (001) facets, and TiO_2_ (100) facets at current density of 50 mA/g in MIBs. **b** Rate performance of anatase TiO_2_, TiO_2_ (001) facets and (100) facets in MIBs. **c** Cycling performances of the TiO_2_ (001) facets at 50 mA/g in MIBs. **d** Charge and discharge curves of the TiO_2_ (001) facets at current densities ranging from 20 mA/g to 1000 mA/g in MIBs. **e** Cycling performances of the TiO_2_ (001) facets at 500 mA/g in MIBs


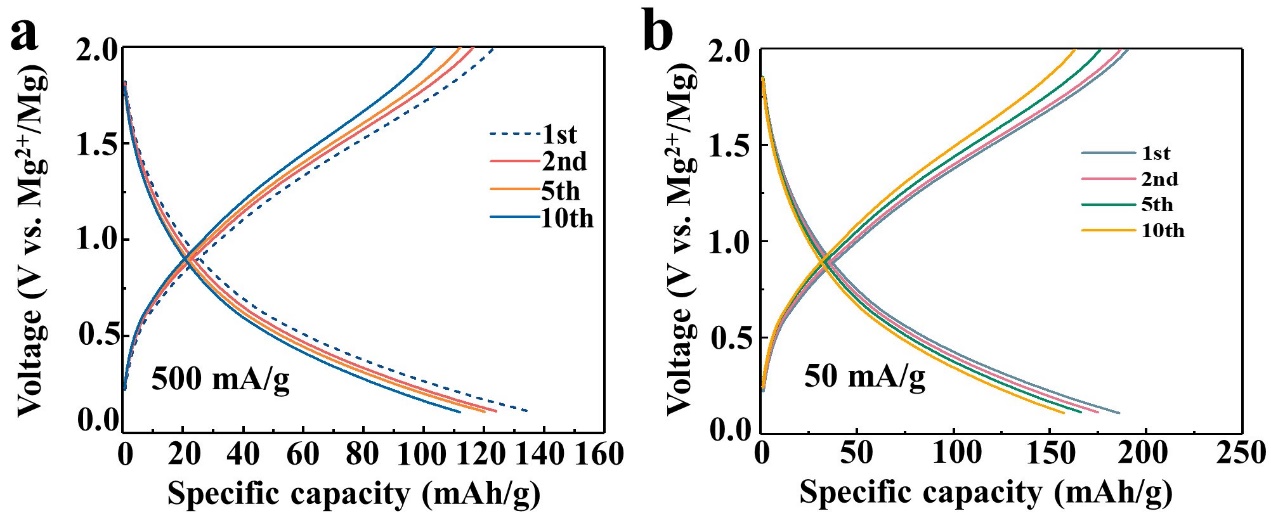


**Fig. S15 a** Voltage profiles of the TiO_2_ (001) facets in MIBs at 500 mA/g and **b** 50 mA/g


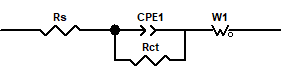


**Fig. S16** The equivalent circuits employed to analyze the EIS curves for the three materials after 20 cycles in the Mg-Li dual-salt electrolyte


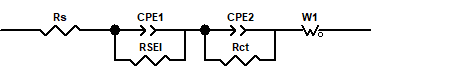


**Fig. S17** The equivalent circuits employed to analyze the EIS curves for the three materials after 20 cycles in the Mg salt electrolyte


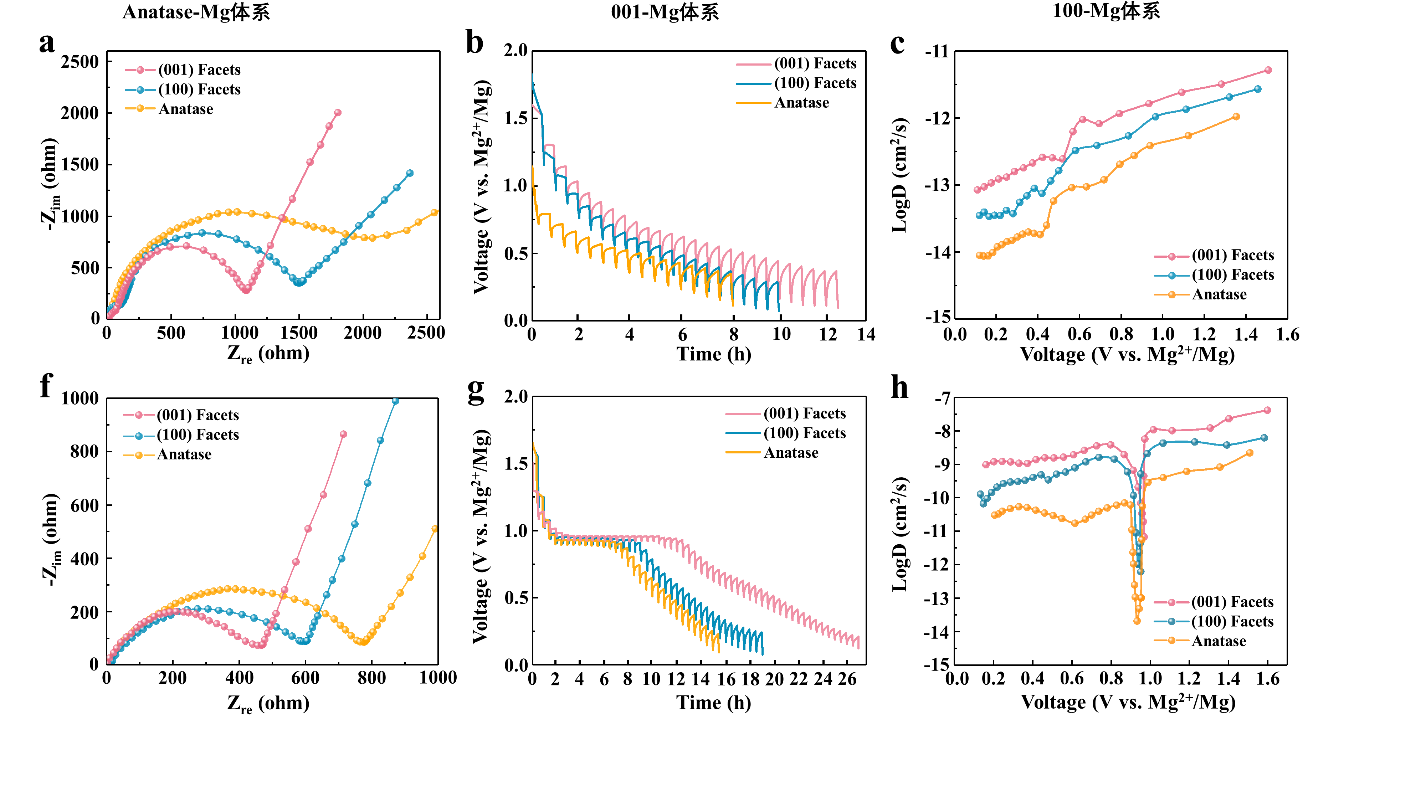


**Fig. S18** **a** Nyquist plots of anatase TiO_2_, TiO_2_ (001) facets and (100) facets in MIBs. **b** GITT profiles of the discharge process of anatase TiO_2_, TiO_2_ (001) facets and (100) facets in MIBs. **c** The diffusion coefficients of ions as a function of the state of discharge process in MIBs


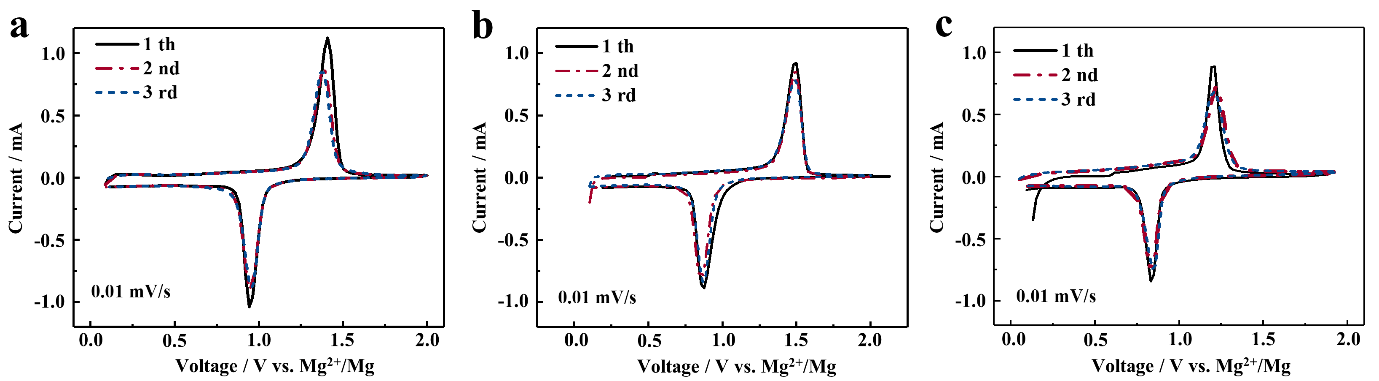


**Fig. S19** CV curves of **a** TiO_2_ (001) facets and **b** TiO_2_ (100) facets and **c** anatase TiO_2_ at 0.01 mV/s in MLIBs


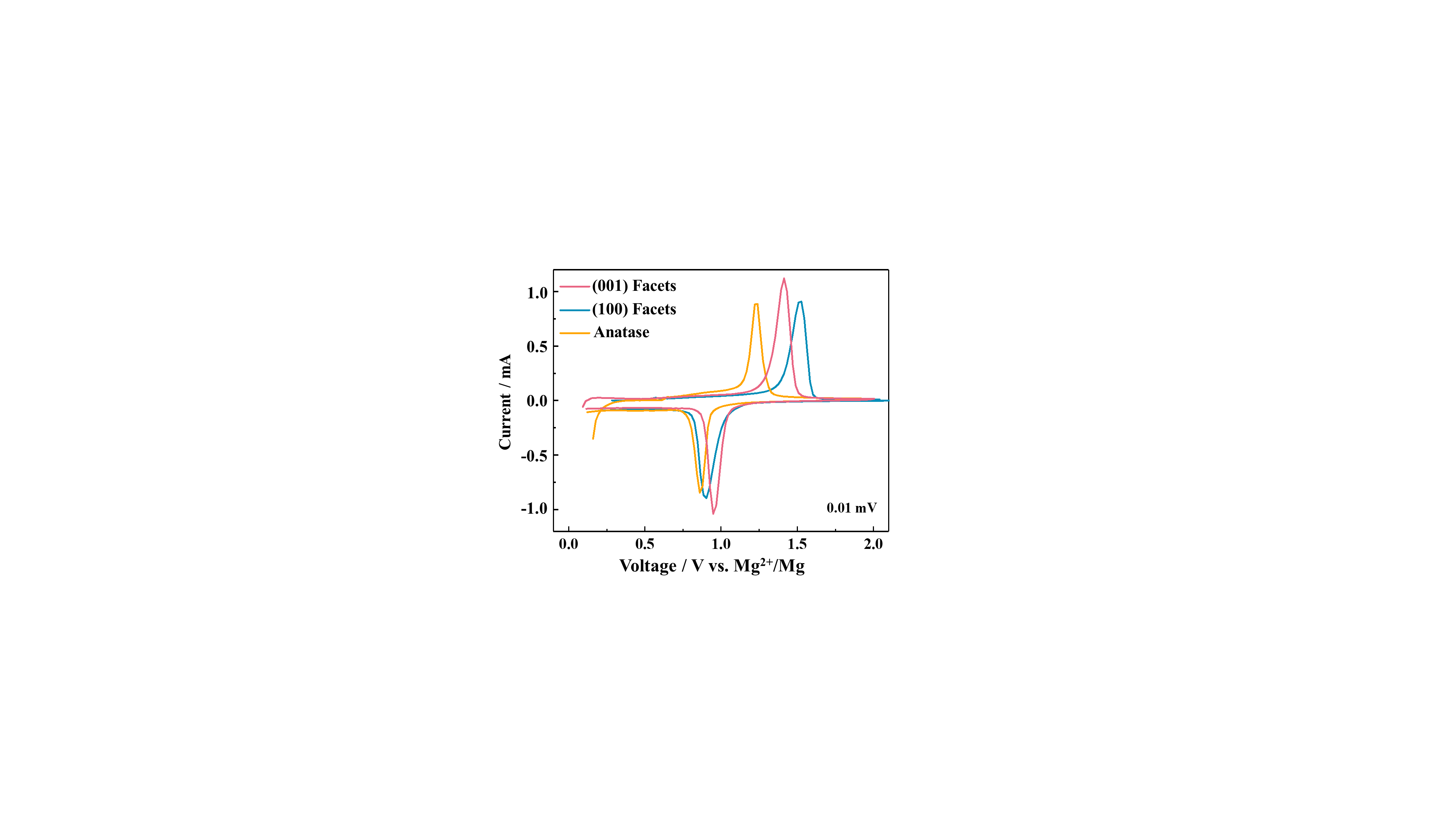


**Fig. S20** CV curves of TiO_2_ (001) facets, TiO_2_ (100) facets and anatase TiO_2_ at 0.01 mV/s in MLIBs


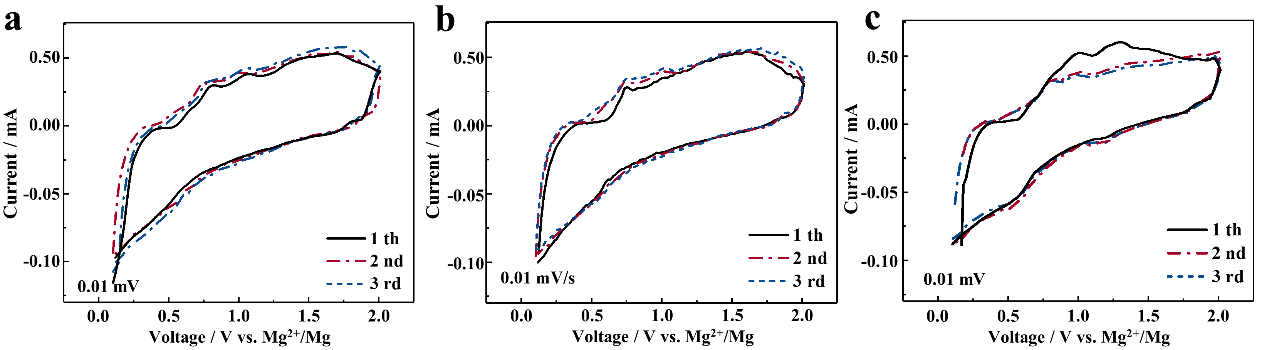


**Fig. S21** CV curves of **a** TiO_2_ (001) facets and **b** TiO_2_ (100) facets and **c** anatase TiO_2_ at 0.01 mV/s in MIBs


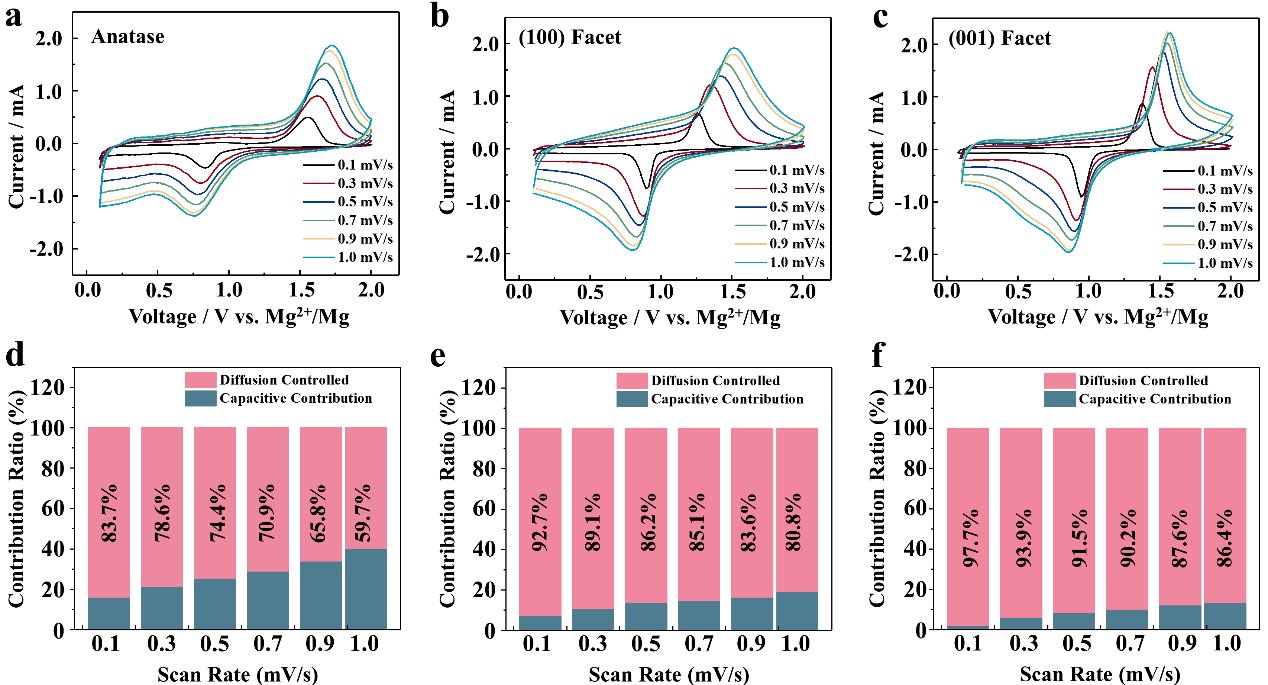


**Fig. S22** **a-c** CV curves of anatase TiO_2_, TiO_2_ (001) facet and (100) facet at different scan rates in MLIBs. **d-f** The diffusion and capacitive contributions of anatase TiO_2_, TiO_2_ (001) facet and (100) facet at different scan rates in MLIBs


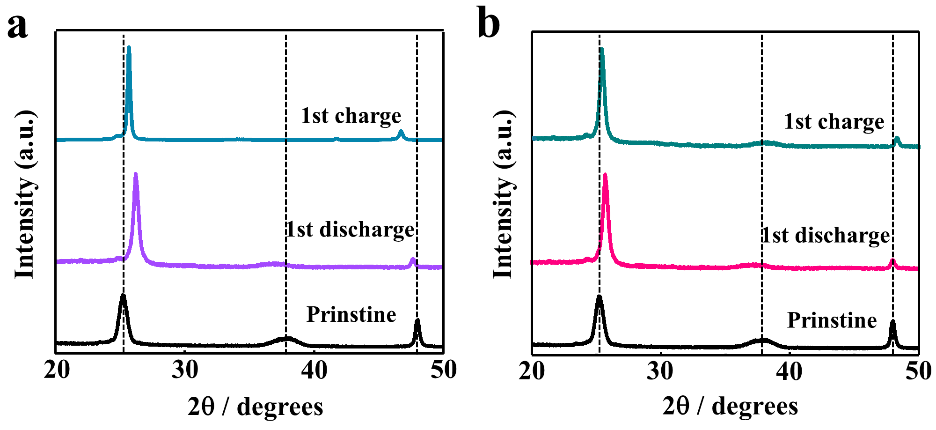


**Fig. S23** *Ex situ* XRD of TiO_2_ (001) facets comparing the **a** Mg-Li system and the reference **b** Mg system


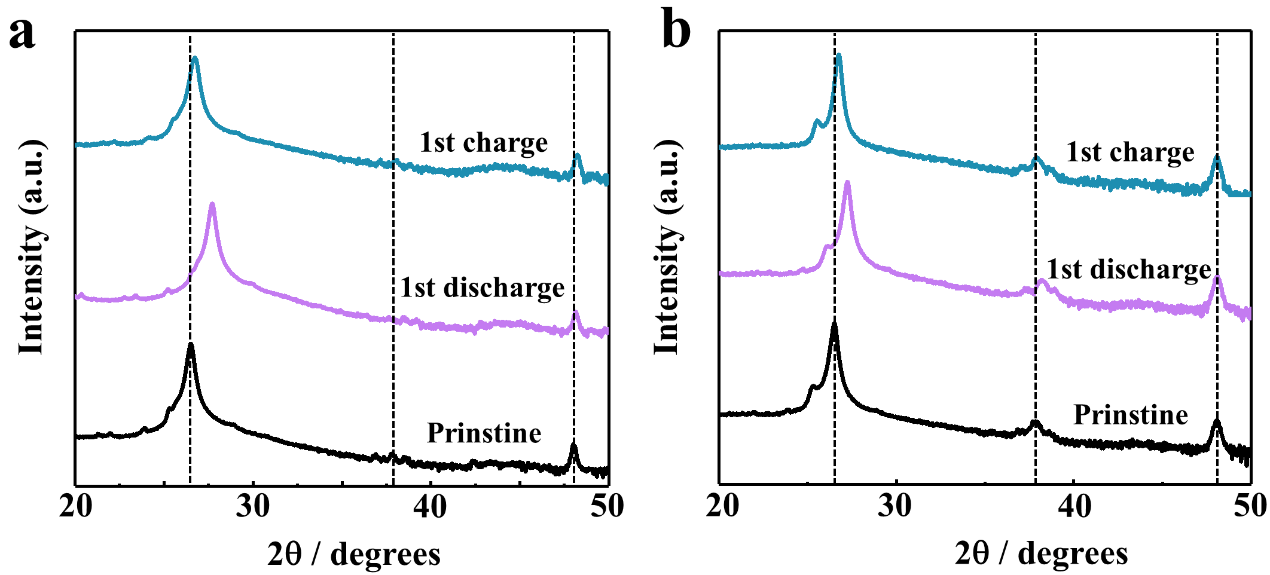


**Fig. S24 a** *Ex situ* XRD of TiO_2_ (100) facets and **b** anatase TiO_2_ comparing the at first discharge and charge states in MLIBs

**
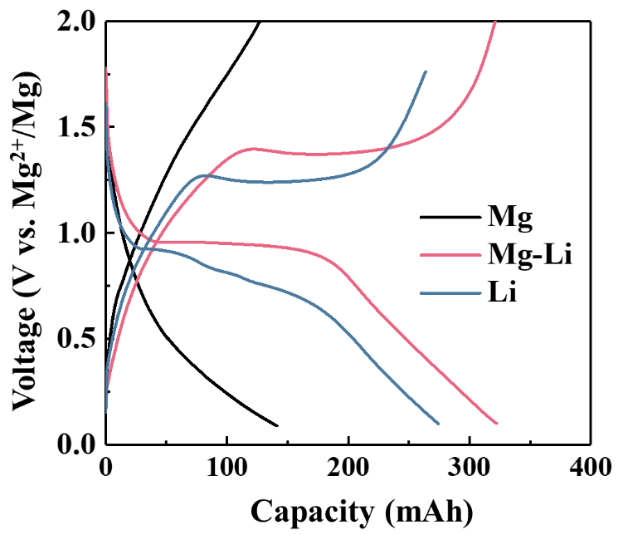
**

**Fig. 25** The GCD curves of TiO₂ (001) material in pure Mg salt, pure Li salt and Mg-Li dual-salt electrolyte


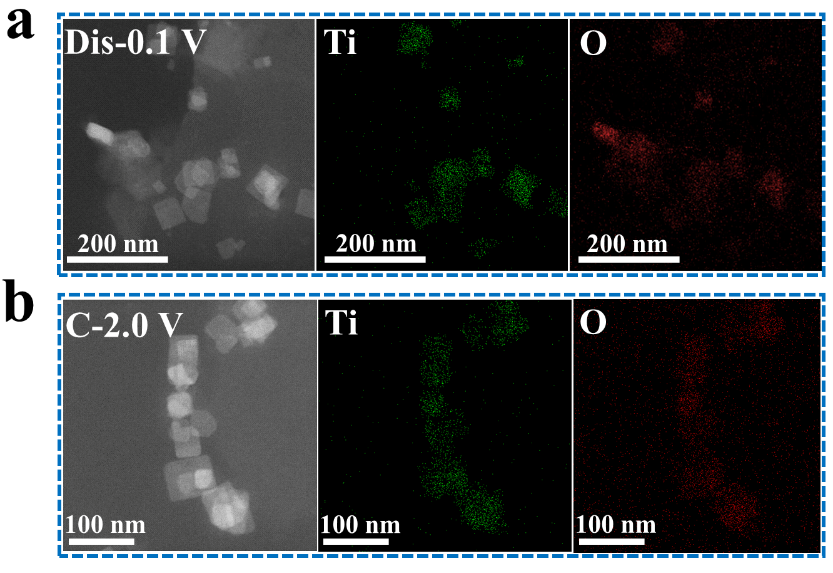


**Fig. S26** **a, b** The TEM and Elemental mapping after 30 cycles of discharging and charging of TiO_2_ (001) facet


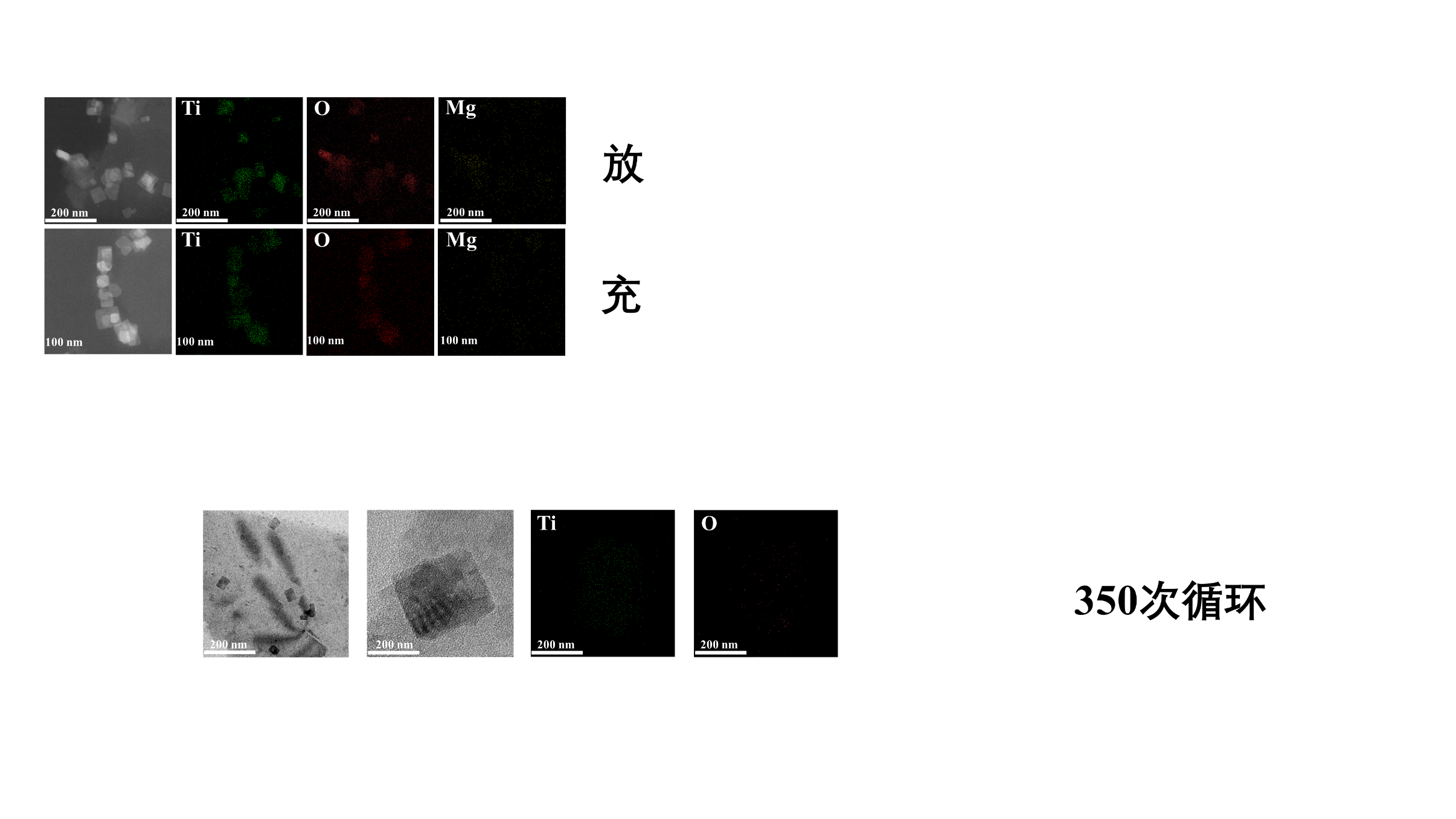


**Fig. S27** The TEM and Elemental mapping after the 350 cycles of discharging and charging of TiO_2_ (001) facet


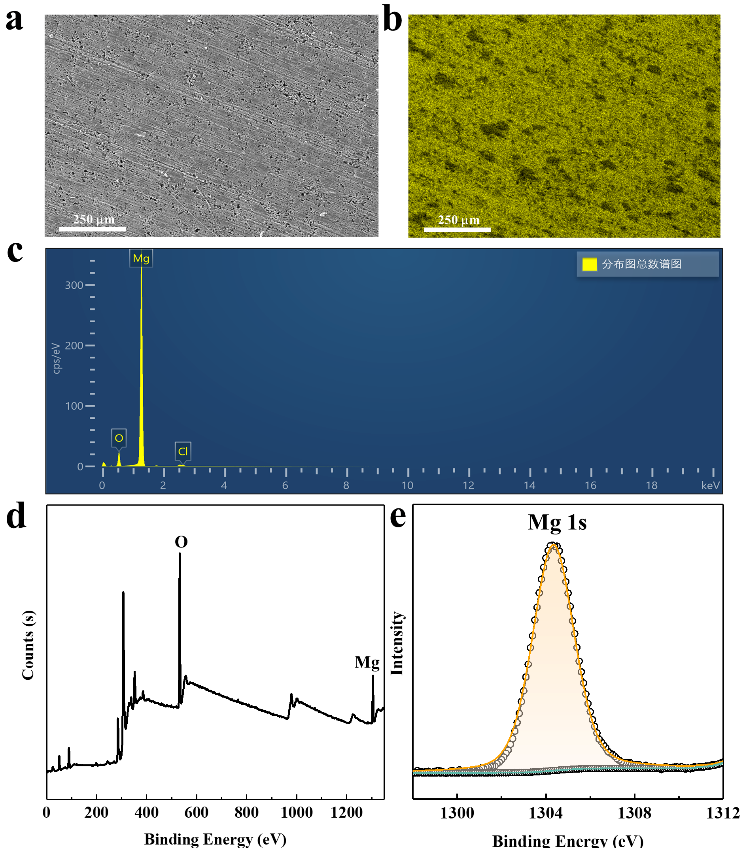


**Fig. S28** **a** SEM image, **b** SEM mapping and **c** EDS of Mg anode. **d-e** XPS spectra of Mg anode


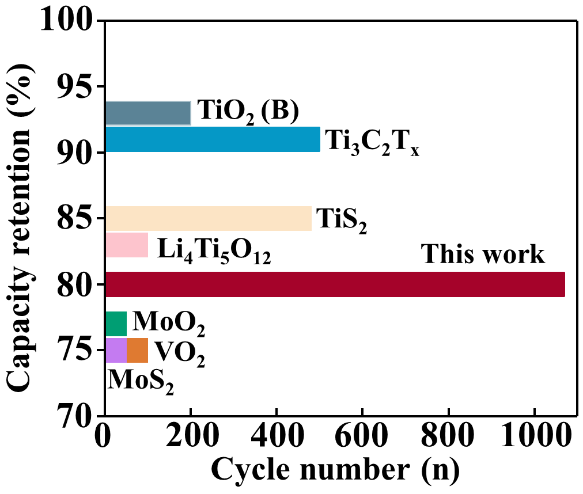


**Fig. 29** The reversible capacity and cycling performance comparisons of TiO_2_ (001) and other typical electrode materials used for MLIBs reported


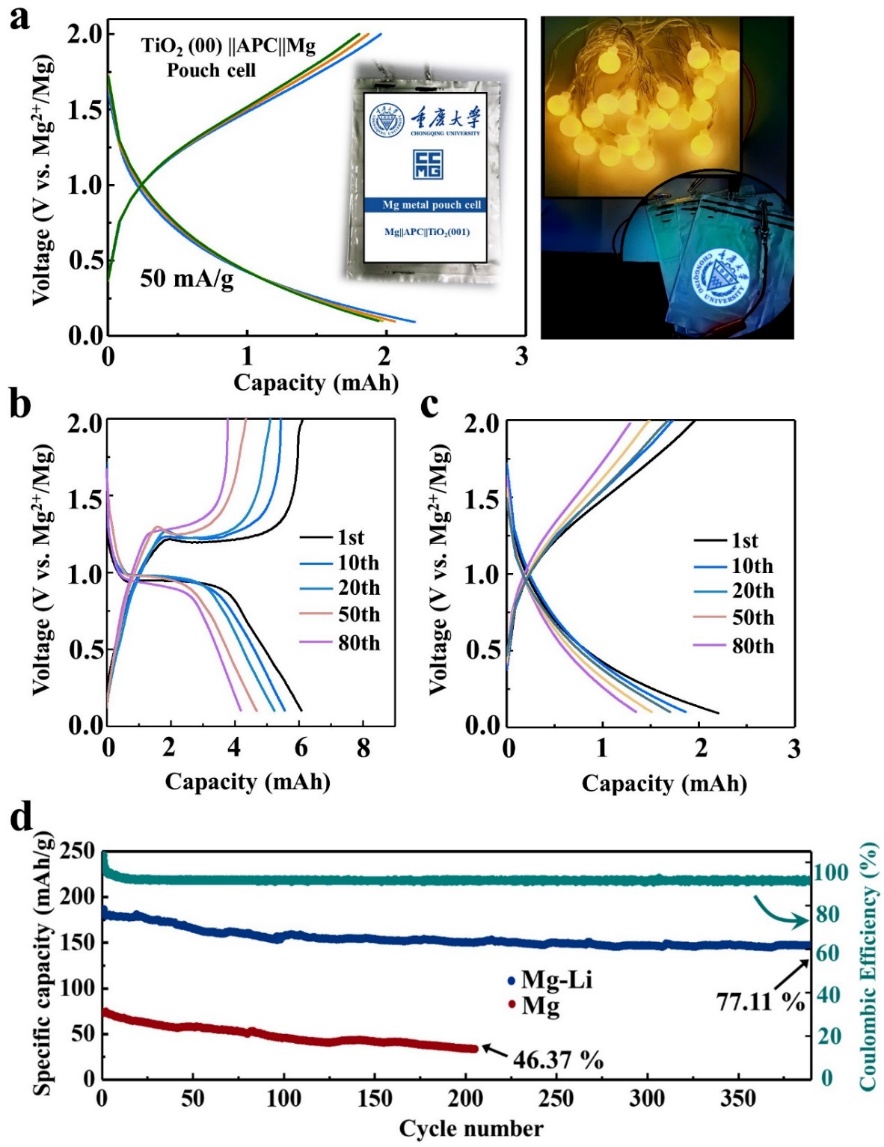


**Fig. S30a** Charge/discharge profiles of the Mg||APC|| TiO_2_ (001) facet pouch cell at 50 mA/g, and the 20 LEDs lightened by two Mg||APC|| TiO_2_ (001) facet pouch cells connected in series. **b V**oltage profiles of the Mg||APC+LiCl|| TiO_2_ (001) facet pouch cell in MLIBs at 50 mA/g. **c** **V**oltage profiles of the Mg||APC|| TiO_2_ (001) facet pouch cell in MIBs at 50 mA/g. **d** Cycling performances of the TiO_2_ (001) facets at 50 mA/g in MIBs and MLIBs

**Table S1** The fitted parameters of the EIS results cycled in Mg-Li dual-salt electrolyte for three materials

| Electrode material | R_ct_ (Ω) |
| --- | --- |
| Anatase TiO_2_ | 766.3 |
| (100) facet | 603.2 |
| (001) facet | 425.8 |

**Table S2** The fitted parameters of the EIS results cycled in Mg salt electrolyte for three materials

| Electrode material | R_ct_ (Ω) | R_SEI_ (Ω) |
| --- | --- | --- |
| Anatase TiO_2_ | 2003.5 | 251.19 |
| (100) facet | 1432.6 | 202.47 |
| (001) facet | 1069.3 | 182.03 |

**Supplementary References**

1. X. Yu, G. Zhao, C. Liu, C. Wu, H. Huang, J. He, N. Zhang, A MoS_2_ and Graphene Alternately Stacking Van Der Waals Heterostructure for Li^+^ /Mg^2+^ Co‐Intercalation. Adv Funct Mater. **31,** (42), 2103214 (2021). <https://doi.org/10.1002/adfm.202103214>
2. F. Liu, Y. Liu, X. Zhao, K. Liu, H. Yin, L.-Z. Fan, Prelithiated V_2_C MXene: A High‐Performance Electrode for Hybrid Magnesium/Lithium‐Ion Batteries by Ion Cointercalation. Small **16,** 1906076 (2020). https://doi.org/ 10.1002/smll.201906076
3. D. Wu, F. Wang, H. Yang, Y. Xu, Y. Zhuang, J. Zeng, Y. Yang, J. Zhao, Realizing Rapid Electrochemical Kinetics of Mg^2+^ in Ti-Nb Oxides through a Li^+^ Intercalation Activated Strategy toward Extremely Fast Charge/Discharge Dual-Ion Batteries. Energy Storage Mater. **52,** 94–103 (2022). <https://doi.org/10.1016/j.ensm.2022.07.042>
4. J. Tian, D. Cao, X. Zhou, J. Hu, M. Huang, C. Li, High-Capacity Mg–Organic Batteries Based on Nanostructured Rhodizonate Salts Activated by Mg–Li Dual-Salt Electrolyte. ACS Nano **12,** 3424–3435 (2018). <https://doi.org/10.1021/acsnano.7b09177>
